# *Cre*-Mediated Stress Affects Sirtuin Expression Levels, Peroxisome Biogenesis and Metabolism, Antioxidant and Proinflammatory Signaling Pathways

**DOI:** 10.1371/journal.pone.0041097

**Published:** 2012-07-19

**Authors:** Yu Xiao, Srikanth Karnati, Guofeng Qian, Anca Nenicu, Wei Fan, Svetlin Tchatalbachev, Anita Höland, Hamid Hossain, Florian Guillou, Georg H. Lüers, Eveline Baumgart-Vogt

**Affiliations:** 1 Institute for Anatomy and Cell Biology II, Justus Liebig University Giessen, Giessen, Germany; 2 Institute for Medical Microbiology, Justus Liebig University Giessen, Giessen, Germany; 3 INRA UMR 85, CNRS UMR 6175, Université François Rabelais de Tours, IFCE Physiologie de la Reproduction et des Comportements, Nouzilly, France; University of Illinois at Chicago, United States of America

## Abstract

*Cre*-mediated excision of *loxP* sites is widely used in mice to manipulate gene function in a tissue-specific manner. To analyze phenotypic alterations related to *Cre*-expression, we have used AMH-*Cre*-transgenic mice as a model system. Different *Cre* expression levels were obtained by investigation of C57BL/6J wild type as well as heterozygous and homozygous AMH-*Cre*-mice. Our results indicate that *Cre-*expression itself in Sertoli cells already has led to oxidative stress and lipid peroxidation (4-HNE lysine adducts), inducing PPARα/γ, peroxisome proliferation and alterations of peroxisome biogenesis (PEX5, PEX13 and PEX14) as well as metabolic proteins (ABCD1, ABCD3, MFP1, thiolase B, catalase). In addition to the strong catalase increase, a NRF2- and FOXO3-mediated antioxidative response (HMOX1 of the endoplasmic reticulum and mitochondrial SOD2) and a NF-κB activation were noted. TGFβ1 and proinflammatory cytokines like IL1, IL6 and TNFα were upregulated and stress-related signaling pathways were induced. Sertoli cell mRNA-microarray analysis revealed an increase of TNFR2-signaling components. 53BP1 recruitment and expression levels for DNA repair genes as well as for p53 were elevated and the ones for related sirtuin deacetylases affected (SIRT 1, 3-7) in Sertoli cells. Under chronic *Cre*-mediated DNA damage conditions a strong downregulation of *Sirt1* was observed, suggesting that the decrease of this important coordinator between DNA repair and metabolic signaling might induce the repression release of major transcription factors regulating metabolic and cytokine-mediated stress pathways. Indeed, caspase-3 was activated and increased germ cell apoptosis was observed, suggesting paracrine effects. In conclusion, the observed wide stress-induced effects and metabolic alterations suggest that it is essential to use the correct control animals (*Cre*/Wt) with matched *Cre* expression levels to differentiate between *Cre*-mediated and specific gene-knock out-mediated effects.

## Introduction

Site specific recombination has become a powerful method for analysis of conditional cell- and tissue-specific gene alterations in mice [1], [2]. One of the integrase family members of site-specific recombinases is the bacteriophage P1 recombinase, *Cre*, which catalyzes recombination between *loxP* DNA elements as a part of the normal viral life cycle [3]. The *Cre*-*loxP* recombination system has become the most widely used systems to delete or to activate gene functions in a tissue-specific or temporally-controlled manner in mice [4], [5], [6].

However, C*re* recombinase is a viral enzyme, and the heterologous expression of this protein has been reported to induce stress and toxic effects in mammalian cells. For example, *Cre* expression has been shown to reduce proliferation and induce chromosomal aberrations related to recombinase activity in cultured cells [7] and can lead to an accumulation of cells in the G2 phase of the cell cycle [8]. Transgenic mice expressing *Cre* under the control of the protamine 1 promoter in spermatids showed postmeiotic chromosomal rearrangements and infertility of male offspring due to arrest in early embryonal development that were related to *Cre*-mediated DNA hydrolysis and/or -ligation of DNA [9]. Furthermore, expression of *Cre* under control of the α-myosin heavy chain promoter induced severe dilatative cardiomyopathy and apoptosis of cardiomyocytes in transgenic mice [10] and the expression of *Cre* under control of a truncated insulin II promoter leads to glucose intolerance in mice [11], indicating that *Cre* expression can cause a phenotype independently of *Cre*-mediated alteration of floxed transgenes. However, despite these reports, the general assumption remains that *Cre* expression does not interfere with physiological functions [4], [5] and does not affect the metabolism of subcellular compartments other than the nucleus. Since our group is working on peroxisomes [12], which are involved in ROS metabolism and adapt their metabolism to various endogenous and exogeneous stimuli [13], we hypothesized that this highly dynamic intracellular compartment could also react to heterologous expression of *Cre*. The spectrum of peroxisomal enzymes involved in ROS metabolism is very wide, comprising catalase and a variety of newly described H_2_O_2_ degrading enzymes, such as glutathione peroxidase I, SOD1, peroxiredoxins I and V and others [14]. In addition, peroxisomes contain various oxidases, generating H_2_O_2_ during the conversion of their metabolic substrate, wherefore they were suggested to be involved in metabolic signaling [15], [16]. Furthermore, peroxisomes harbor two beta-oxidation systems, metabolizing very long-chain fatty acids, bioactive and signaling lipids (arachidonic acid, eicosanoids, n-3 and n-6 fatty acids) as well as enzymes for the synthesis of polyunsaturated fatty acids (PUFA), plasmalogens and cholesterol [17], [18]. Peroxisomal lipid metabolites are ligands for nuclear receptors of the PPAR-family (PPARα, β, γ). Since PPARs increase the transcription of genes for peroxisomal β-oxidation enzymes, these organelles might be important regulators for the homeostasis of the lipid ligands binding to nuclear receptors [19]. Furthermore, due to the generation of H_2_O_2_ by the function of acyl-CoA oxidases and the ROS-trapping activity of PUFA and plasmalogens, peroxisomes and their metabolites link intracellular ROS- and lipid metabolism to each other [16].

Finally, genes involved in the biogenesis of peroxisomes (*Pex* genes encoding peroxins) are induced by ROS in plants and mammalian cells [14], [20]. To date, 32 distinct peroxin proteins have been described, which are located either in the cytoplasm, on the peroxisomal membrane, or inside of the peroxisomal matrix [21]. Knockout of *Pex* genes in mice leads to a disruption of all peroxisomal metabolic pathways [22], combined with secondary effects on mitochondria [23] and results in a similar phenotype to the Zellweger syndrome in human patients with peroxisomal disorders [24], [25]. However, because these knockout animals die within the first day of life, conditional knockouts with the *Cre*-recombinase technology were used in recent years to study the metabolic role of these organelles in different organ systems [26].

Since we were interested in the functions and heterogeneities of peroxisomes in the testis [27], [28], [29] and in their relevance for fertility, we have started investigations with conditional inactivation of *Pex13* in different cell types of the testis. For this purpose, we have initiated studies with transgenic mice that express the *Cre* recombinase under the control of the anti-Müllerian hormone (AMH) promoter [30], [31] to restrict the *Cre* expression to Sertoli cells. Already in preliminary analyses of these mice, we have noted that sole *Cre*-expression leads indeed to disturbances in metabolic pathways of peroxisomes and other cell organelles and have set up to analyze these alterations in details. Alterations induced by sole *Cre* expression might essentially influence the interpretation of knockout mouse phenotypes, especially when promoters with high activity are used to drive the *Cre* expression in different tissues (e.g. myosin-*Cre* for heart, insulin-*Cre* for β-cells in the pancreas, albumin-*Cre* for hepatocytes in the liver, etc…), since the correct *Cre/*Wt controls that are necessary for accurate interpretation of results can only be generated for knockout models with autosomal-recessive inheritance in separate matings. Many groups in the literature, however, did not include this control in their articles. *Cre*-mediated secondary effects on organelles and stress pathways, however, might have a strong impact on the interpretation of research data concerning the phenotypic characterization of knockout animals.

Therefore, we have analyzed these aspects in our present study and show that sole expression of *Cre-*recombinase in Sertoli cells causes significant alterations in peroxisomal ROS- and lipid metabolism as well as general antioxidant and proinflammatory signaling pathways and synthesis of paracrine mediators. The observed severe alterations of expression of sirtuin proteins in this study could explain why DNA damage exerted by the *Cre* recombinase could lead to induction of stress pathways and strong metabolic alterations in the testis, leading through paracrine effects in consequence to germ cell apoptosis.

## Materials and Methods

### Mice – Ethics Statement

Breeding and handling of AMH-*Cre*-transgenic mice was approved by the Governmental Commission of Animal Care (ethics committee: Regierungspraesidium Giessen, Germany, permit number: V 54-19 C 20/15 c GI 20/23). Animals were housed under standard conditions (12 h light and 12 h dark cycle) with free access to food and water in the central animal facility (Zentrales Tierlabor - ZTL) of the Justus Liebig University (Giessen, Germany). Heterozygous AMH-*Cre*/Wt mice were generated and described by Lécureuil and colleagues [30]. These mice were crossed and off-springs (homozygous AMH-*Cre*/AMH-*Cre*, heterozygous AMH-*Cre*/Wt and Wt/Wt) were used at day P14 or P21 for our experiments (additional information on the testis of P120 day animals was added in Figure S4).

### Localization of the AMH-*Cre* Transgene in the Genome by PCR-based Genome Walking

**Figure 1:**
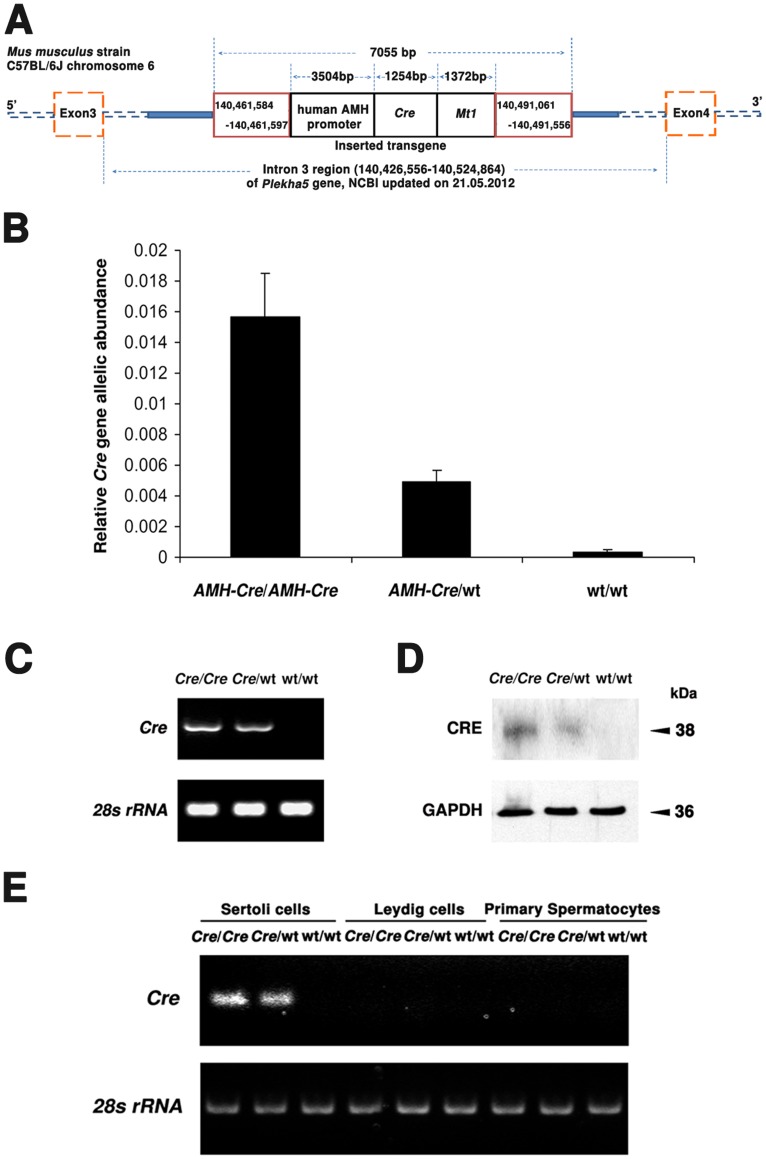
*Cre* expression in the testis correlates with allelic abundance/genotype. **A**) The consensus and integrity of inserted AMH-*Cre* transgene (black box) and flanking regions (red box) of *Mus musculus* strain C57BL/6J was confirmed by PCR-based genome walking. **B**) Genotyping on genomic tail DNA using quantitative real-time PCR. Values were normalized for *28s rRNA* and are given as relative *Cre* transgene allelic abundance (average ± standard deviation). **C**) Semiquantitative RT-PCR analyses for the *Cre* expresssion and *28s rRNA* on cDNAs prepared from total testicular RNA (genotypes are indicated). **D**) Western blot analysis of *Cre* and GAPDH abundance in the testis from wild type and *Cre* transgenic mice. 20 µg of protein were loaded per lane. **E**) Semiquantitative RT-PCR analyses for *Cre* expression and *28s rRNA* on cDNAs from total RNA of microdissection samples of Sertoli cells, Leydig cells and primary spermatocytes in the testis from wild type and *Cre* transgenic mice.

Ten µg genomic DNA was isolated from the tails of AMH-*Cre*/AMH-*Cre* mice using the Wizard® SV Genomic DNA Purification System (Promega, Mannheim, Germany). For sequencing an improved method for PCR-based genome walking in uncloned DNA [32] was applied to characterize the exact insertion and the flanking region of the AMH-*Cre* transgene cassette into the mouse genome. The transgene cassette, driving the *Cre* gene under the control of the human AMH-promoter (HsAMH promoter-*Cre-Mt1*), was derived in part from the pBS185 *CMV-Cre* plasmid from the “addgene” plasmid repository (Cambridge, MA 02139, USA) and was described by Lécureuil and colleagues (2002) (Fig. 1 in [30]). The sequencing experiments were performed by Beckman Coulter Genomics Inc. (36 Cherry Hill Drive, Danvers, MA 01923, USA). The total sequence of the transgenic insertion and flanking regions is provided in Sequence S1; the recombinant allele containing the transgene cassette is depicted in Fig. 1A.

### Genotyping by Real-time PCR

Genomic DNA was isolated from mouse tail biopsies [33]. The concentration of isolated DNA was measured using a NanoDrop® ND-1000 UV-Vis spectrophotometer (NanoDrop Technologies, Rockland, USA). The final concentration of every DNA sample was adjusted to 400 ng/µl. Real-time PCR was performed with the iQ SYBR Green Supermix in an iCycler according to the manufacturer’s suggestions (Bio-Rad, Munich, Germany). The following primers were used: *Cre*-forward primer 5′-GCATTTCTGGGGATTGCTTA-3′, *Cre*-reverse primer 5′-ATTCTCCCACCGTCAGTACG-3′, yielding a product of 95 bp; *28s rRNA*-forward primer 5′-AAAGCGGGTGGTAAACTCCA-3′, *28s rRNA*-reverse primer 5′-GGTTTCACGCCCTCTTGAAC-3′, yielding a product of 118 bp. PCR conditions used were: denaturation at 95°C for 8 min, followed by 45 cycles of denaturation at 95°C for 20 s, annealing at 62°C for 30 s and elongation at 72°C for 30 s. DNA samples of three different experiments were analyzed in triplicate. Values were normalized for the abundance of the amplified *28s rRNA* alleles in each experiment. Data are presented as the relative *Cre* transgene allelic abundance = 2^−Δct^, Δct = ct*_Cre_* – ct*_28s rRNA_* (Ct = threshold cycle).

### RNA Expression Analyses

#### RNA isolation from complete testes

Total RNA was isolated from frozen testis using the RNeasy Mini Kit from Qiagen, according to the manufacturer’s protocol (Qiagen, Hilden, Germany). Each RNA preparation was subjected to DNase I digestion to remove possible contamination of genomic DNA. The quantity and integrity of the isolated RNA was assessed with a NanoDrop® ND-1000 UV-Vis spectrophotometer (NanoDrop Technologies, Rockland, USA) and an Agilent 2100 bioanalyzer (Agilent Technologies, Waldbronn, Germany).

#### Laser capture microdissection to isolate RNA from specific testicular cell types

Nine µm thick cryosections were cut from Tissue-Tek®-embedded (Sakura Finetek Europe B.V., Zoeterwoude, The Netherlands) frozen mouse testes samples with a Leica CM3050 cryomicrotome. Sections were mounted on nuclease free glass slides (Carl Zeiss MicroImaging GmbH, Munich, Germany), air dried for 10 min and subsequently fixed with 70% and 90% ethanol for 1 min each. Sections were stained with H&E (1 min Mayer’s hematoxylin solution, 1 min eosin solution), washed with 100% ethanol for 1 min and air dried for 2 min. After staining, the sections were immediately used for laser capture microdissection. Sertoli cells, Leydig cells and primary spermatogonia were cut out using an Axiovert microscope (AxioCam ICc 1) equipped with a P.A.L.M. Laser Capture Microdissection system (Carl Zeiss MicroImaging GmbH, Munich, Germany). About 100 cells of each cell type were collected in separate nuclease free tubes and suspended in 300 µl RLT buffer (Qiagen, Hilden, Germany), supplemented with 10% β-mercaptoethanol. The RNA from every sample was isolated using the Qiagen Micro kit (Qiagen, Hilden, Germany) according to the manufacturer’s protocol.

#### Reverse transcription and RT-PCR

First-strand cDNA was synthesized from 1 µg of total RNA with oligo (dT) 12–18 primers using Superscript II reverse transcriptase (Invitrogen, Karlsruhe, Germany). For the polymerase chain reactions (PCR) 400 ng cDNA per 25 µl reaction were used. All primer pairs were tested for optimal annealing temperatures and PCR conditions (26, 29 or 32 cycles) were optimized with gradient PCRs on a Bio-Rad iCycler. Primer sequences and annealing temperatures are summarized in Table 1. Bands on agarose gels were quantified with a Bio-Rad Gel Doc 2000 system (Bio-Rad, Munich, Germany). All RT-PCR experiments were performed three times and represent data from three individual mice for every genotype. For space reasons, representative PCR samples of each genotype were run on a second agarose gel and photographed with a Bio-Rad gel documentation system.

**Table 1:** List of primers for RT-PCR.

Gene	Symbol	Forward primer (5′-3′)	Reverse primer (5′-3′)	Annealing Temperature (°C)	Length (bp)
3-ketoacyl-CoA thiolase B	*Thiolase*	5′-TACGGTGAGTGATGGAGCAG-3′	5′-CACACAGTAGACGGCCTGAC-3′	65	238
Active regulator of SIRT1	*Aros*	5′-CATTGTCGAGACCACCTCAA-3′	5′-GATGGTTCCGCCAAACTAGA-3′	59	458
Alkylglycerone phosphatesynthase	*Agps*	5′-TTTTGGGAAACAAAAGCTCAA-3′	5′-TTGGAGCAACACACTTCAGG-3′	56	250
Ataxia telangiectasia mutatedhomolog	*Atm*	5′-GTGTGGCATTGATCTCACTGAGG-3′	5′-CTCAAATGTGTGCTTTGCCTAGC-3′	62	574
ATP-binding cassette transporter,sub-family D, member 1,peroxisomal membrane protein	*Abcd1*	5′-GAGGGAGGTTGGGAGGCAGT-3′	5′-GGTGGGAGCTGGGGATAAGG-3′	67	465
ATP-binding cassette transporter,sub-family D, member 3,peroxisomal membrane protein	*Abcd3*	5′-CTGGGCGTGAAATGACTAGATTGG-3′	5′-AGCTGCACATTGTCCAAGTACTCC-3′	65	523
BCL2-associated agonist of celldeath	*Bad*	5′-AGGACTTATCAGCCGAAGCA-3′	5′-GCTAAGCTCCTCCTCCATCC-3′	61	420
Catalase	*Cat*	5′-ATGGTCTGGGACTTCTGGAGTCTTC-3′	5′-GTTTCCTCTCCTCCTCATTCAACAC-3′	64	833
Cyclization recombinase	*Cre*	5′-CACCCTGTTACGTATAGC-3′	5′-CTAATCGCCATCTTCCAG-3′	55	526
Cyclooxygenase-2	*Cox2*	5′-TGCATGTGGCTGTGGATGTCATCA-3′	5′-CACTAAGACAGACCCGTCATCTCCA-3′	66	449
DNA cross-link repair 1C,PSO2 homolog	*Artemis*	5′-CCTGAGTGGCAGGAAGTCTC-3′	5′-CAGGCCACATACATCCACAG-3′	61	379
Deleted in bladder cancer 1	*Dbc1*	5′-ATCCACATGGTGATCGGAAT-3′	5′-CAGGCTGTACCCAAACACCT-3′	58	372
Enoyl-Coenzyme A, hydratase	*Mfp1*	5′-ATGGCCAGATTTCAGGAATG-3′	5′-TGCCACTTTTGTTGATTTGC-3′	60	211
Follicle stimulating hormonereceptor	*Fshr*	5′-CCAGCCTTACCTACCCCAGT-3′	5′-CTGTGGTGTTCCCAGTGATG-3′	62	345
Forkhead box O1	*Foxo1*	5′-GGGAGAATGTTCGCTTTCTGGT-3′	5′-CCATCCTCATCAGGCACTTCTC-3′	61	662
Forkhead box O3	*Foxo3*	5′-CATCTCAAAGCTGGGTACCAGG-3′	5′-GCTCAAGGCCAGACTGGGAACT-3′	63	541
Forkhead box O4	*Foxo4*	5′-TTGTGCCTAGAAGAGAGTGCTG-3′	5′-ATTTGACACACTTCCCCACTCC-3′	60	531
GATA binding protein 4	*Gata4*	5′-CGGGTTGTTCAAACACCTTT-3′	5′-TGTGTGTGAAGGGGTGAAAA-3′	58	617
Glutathione peroxidase 1	*Gpx1*	5′-GGGACTACACCGAGATGAACGA-3′	5′-ACCATTCACTTCGCACTTCTCA-3′	61	197
Glutathione S-transferase, alpha 1	*Gsta1*	5′-GCAGACCAGAGCCATTCTCAACTAC-3′	5′-CTGCCAGGCTGTAGGAACTTCTTC-3′	55	408
Glyceraldehyde-3-phosphatedehydrogenase	*Gapdh*	5′-CACCATGGAGAAGGCCGGGG-3′	5′-GACGGACACATTGGGGGTAG-3′	60	391
GlyceronephosphateO-acyltransferase	*Gnpat*	5′-CCGTCTCCTTGAGACCTCTG-3′	5′-AGGTGTGGGAATCTGAGTGG-3′	60	198
Heme oxygenase-1	*Hmox1*	5′-GCACTATGTAAAGCGTCTCCACGAG-3′	5′-CCAGGCAAGATTCTCCCTTACAGAG-3′	65	610
Inhibin alpha	*Inha*	5′-GCAATGGATGGGGAAGGTGG-3′	5′-GGTGGCTGCGTATGTGTTGG-3′	65	238
Inhibitor of kappaB kinase beta	*Ikbkb*	5′-CAGGACACTGTGAAGGAGCA-3′	5′-TTGTACAGCAGCCATGAAGC-3′	59	306
Inhibitor of kappaB kinase gamma	*Ikbkg*	5′-TGCCTTCAGAGCAGGGTACT-3′	5′-CTAAAGCTTGCCGATCCTG-3′	59	390
Interleukin 1 alpha	*Il1α*	5′-CGTCAGGCAGAAGTTTGTCA-3′	5′-GTGCAAGTGACTCAGGGTGA-3′	61	516
Interleukin 1 beta	*Il1β*	5′-GGACATGAGCACCTTCTTTTCC-3′	5′-GTGCCGTCTTTCATTACACAGG-3′	63	321
Interleukin 6	*Il6*	5′-GTTCTCTGGGAAATCGTGGA-3′	5′-GGAAATTGGGGTAGGAAGGA-3′	61	339
Isopentenyl-diphosphatedelta isomerase	*Idi1*	5′-TCTGTCTCGGTCTTCAGACAGGATG-3′	5′-AGTACCTGGGAGTCAGAGGAAGGTG-3′	67	718
Luteinizing hormone/choriogonadotropin receptor	*Lhcgr*	5′-ACCAAAAGCTGAGGCTGAGA-3′	5′-TACAGAAATGGGTTGGCACA-3′	66	445
Meiotic recombination11 homolog A	*Mre11*	5′-TGATGACGATGACCCTTTCA-3′	5′-ACAACGGAGGGGTTTTCTCT-3′	57	368
Mitogen-activated protein 4kinase 4	*Nik*	5′-GACAGCAGGGAGTAGCCTTG-3′	5′-GGAGAAAAGCCAGAGTGTCG-3′	60	346
Nibrin	*Nbs1*	5′-TTCCTGCACGTTGAAGACAG-3′	5′-CTTGTGCTGCTTGGCATTTA-3′	57	448
Nitric oxide synthase 2, inducible	*Nos2*	5′-GTGTTCCACCAGGAGATGTTG-3′	5′-CTCCTGCCCACTGAGTTCGTC-3′	66	576
Nuclear factor erythroid 2-relatedfactor	*Nrf2*	5′-CCACTGGTTTAGCCATCTCTCC-3′	5′-GTGGACATTAGCCCTTCCAAAC-3′	64	364
Nuclear factor NF-kappa-B p65 subunit	*Rela*	5′-GGCCTCATCCACATGAACTT-3′	5′-ATCTTGAGCTCGGCAGTGTT-3′	57	337
Peroxisome biogenesis factor 5	*Pex5*	5′-GAGTGAAGAAGCAGTGGCTGCATAC-3′	5′-GGACAGAGACAGCTCATCCCTACAA-3′	64	508
Peroxisome biogenesis factor 11 alpha	*Pex11α*	5′-TCAGCTGCTGTGTTCTCAGTCCTT-3′	5′-GTACTTAGGAGGGTCCCGAGAGGA-3′	64	420
Peroxisome biogenesis factor 11 beta	*Pex11β*	5′-GTATGCCTGTTCCCTTCTCG-3′	5′-CTCGGTTGAGGTGACTGACA-3′	65	216
Peroxisome biogenesis factor 11 gamma	*Pex11γ*	5′-GACTCTGCTTGGTGGTGGACACT-3′	5′-TGTCTCTCCCACTCACCTTTAGGC-3′	64	682
Peroxisome biogenesis factor 13	*Pex13*	5′-GACCACGTAGTTGCAAGAGCAGAGT-3′	5′-CTGAGGCAGCTTGTGTGTTCTACTG-3′	65	718
Peroxisome biogenesis factor 14	*Pex14*	5′-CACCTCACTCCGCAGCCATA-3′	5′-AGGATGAGGGGCAGCAGGTA -3′	60	131
Peroxisome proliferator activated receptor alpha	*Pparα*	5′-AGACCGTCACGGAGCTCACA-3′	5′-GGCCTGCCATCTCAGGAAAG-3′	68	584
Peroxisome proliferator activated receptor beta	*Pparβ*	5′-CACCGAGTTCGCCAAGAACA-3′	5′-AGAGCCCGCAGAATGGTGTC-3′	60	363
Peroxisome proliferator activated receptor gamma	*Pparγ*	5′-TCCGTAGAAGCCGTGCAAGA-3′	5′-CACCTTGGCGAACAGCTGAG-3′	60	441
Peroxisome proliferative activated receptor, gamma, coactivator 1 alpha	*Pgc1α*	5′-ATGTGTCGCCTTCTTGCTCT-3′	5′-GCGGTATTCATCCCTCTTGA-3′	57	350
Poly (ADP-ribose) polymerase family, member 1	*Parp1*	5′-TGAGAAACTCGGAGGCAAGT-3′	5′-TGCAGAGTGTTCCAGACCAG-3′	59	391
Sirtuin 1	*Sirt1*	5′-GGTAGAGCCTGCATAGATCTTCA-3′	5′-TGGCAGTAATGGTCCTAACTGGG-3′	62	512
Sirtuin 2	*Sirt2*	5′-CCATGACCTCCCGCAGGACAGCG-3′	5′-GGGTCCCCAGGAAAGGGAGCCTA-3′	69	505
Sirtuin 3	*Sirt3*	5′-GTGCCCCGACTGCTCATCAATCG-3′	5′-CCGATCAACATGCTAGATTGCCC-3′	65	523
Sirtuin 4	*Sirt4*	5′-CACCCGGTCTGACGATTTGGCTT-3′	5′-CCGTGTTAGCTATTGCTCCTGCC-3′	64	483
Sirtuin 5	*Sirt5*	5′-TTGCTGCGACCTCACGTGGTGTG-3′	5′-GGAAGGACTTGACAGCCTCTTCC-3′	65	505
Sirtuin 6	*Sirt6*	5′-GGGGACTGAGCCCAGGTTTGCAT-3′	5′-CTTCTGGGAGCCTGGGGCCCTTA-3′	67	494
Sirtuin 7	*Sirt7*	5′-TATCCTAGGAGGCTGGTTTGGCA-3′	5′-GGAGGCTTAGTTAGATTCTCCCT-3′	59	503
Superoxide dismutase 1, soluble	*Sod1*	5′-TGGGTTCCACGTCCATCAG-3′	5′-ACACCGTCCTTTCCAGCAG-3′	63	153
Superoxide dismutase 2, mitochondrial	*Sod2*	5′-ATGCAGCTGCACCACAGCAA-3′	5′-ACTTCAGTGCAGGCTGAAGAG-3′	64	130
TNF receptor-associated factor 2	*Traf2*	5′-CCAGTGACCACTCCATTGTG-3′	5′-GAGAAGCCATGAGCACAACA-3′	59	341
TRAF family member-associated NF-kappa B activator	*Tank*	5′-AGCCCAGGCTAAAGATGAT-3′	5′-TTATGGGGTCAATTCCCTGA-3′	58	306
Transferrin	*Trf*	5′-ATCTGGGAGATTCTCAAAGTG-3′	5′-AGTGTGGCAGGACTTCTTGCC-3′	64	579
Transforming growth factor, beta 1	*Tgfβ1*	5′-GAGCCACAAACCCCGCCTCC-3′	5′-GGGGTTCGGGCACTGCTTCC-3′	66	860
Transforming growth factor, beta 2	*Tgfβ2*	5′-GCCCCCGGACCTTCTCGTCT-3′	5′-CGGCCACTCTGGCTTTGGGG-3′	66	924
Transforming growth factor, beta 3	*Tgfβ3*	5′-CAGCTGCCTTGCCACCCCTC-3′	5′-CCTCAACAGCCACTCGCGCA-3′	65	966
Transformation related protein 53	*p53*	5′-AGAGACCGCCGTACAGAAGA-3′	5′-TATGGCGGGAAGTAGACTGG-3′	59	304
Tumor necrosis factor	*Tnf*	5′-GGCCTCCCTCTCATCAGTTCTA-3′	5′-GGGTCAGAGTAAAGGGGTCAGA-3′	62	571
Tumor necrosis factor receptor 1a	*Tnfr1*	5′-CTGTATGCTGTGGTGGATGG-3′	5′-TTTCACCCACAGGGAGTAGG-3′	59	371
Tumor necrosis factor receptor 1b	*Tnfr2*	5′-TGTAGCCTGTGGATGCTGAG-3′	5′-CATGCAAACATGGACACACA-3′	58	324
X-ray repair complementing defective repair in Chinese hamster cells 1	*Xrcc1*	5′-TCTTCTCAAGGCGGACACTT-3′	5′-GCCTCTGCCTCATCTTTGTC-3′	59	417
28S ribosomal RNA	*28s rRNA*	5′-AAAGCGGGTGGTAAACTCCA-3′	5′-GGTTTCACGCCCTCTTGAAC-3′	62	118

#### Sertoli cell cultures of individual mice (AMH-*Cre*/Wt and Wt/Wt) for total RNA isolation

Sertoli cells from individual 14-day-old mice were isolated as described by Nenicu et al [28] with small adaptations for the isolation of the cells from individual animals. Decapsulated testes were minced into small fragments and incubated with 1 mg/ml collagenase A, 15 mM HEPES and 20 µg/ml DNase in DMEM/F12 for 15 min at 37°C in a shaking water bath incubator, followed by a 15 min sedimentation step at room temperature. After sedimentation the supernatant was removed and the cells were dispersed in the same solution containing additional 2 mg/ml hyaluronidase for 30 min at 34°C. The digestion was stopped by centrifuging the cell suspension for 45 s at 500 rpm and resuspension in DMEM/F12, supplemented with soybean trypsin inhibitor (400 µg/ml) and BSA (2 mg/ml). The cell suspension was recentrifuged for 60 s at 500 rpm and the supernatant was discarded. The cell pellet was dispersed and further digested with 2 mg/ml collagenase A, 2 mg/ml hyaluronidase and 20 µg/ml DNase in DMEM/F12 for 20 min at 34°C, followed by centrifugation for 45 s at 1,000 rpm. The pellet was resuspended in DMEM/F12 and recentrifuged for 60 s at 1,000 rpm. Thereafter, the cells were resuspended in 15 ml DMEM/F12 supplemented with 2 mM L-glutamine, 100 U/ml penicillin, 100 µg/ml streptomycin, 10 ng/ml epidermal growth factor, 5 µg/ml human transferrin, 2 µg/ml insulin, 10 nM testosterone, 100 ng/ml follicle-stimulating hormone (FSH) and 3 ng/ml cytosine arabinoside. Cell clusters were gently dispersed by homogenization with a potter (10 strokes) and the cells filtered through a sterile nylon mash (70 µm pore size, BD Falcon), followed by centrifugation for 2 min at 800 rpm. The upper 5 ml of the tube were discarded and the remaining 10 ml were mixed and transferred into 1 well (9.6 cm^2^) of a matrigel-coated 6-well plate for each animal preparation and incubated at 34°C in a humidified atmosphere with 5% CO2. After 3 days of culture they were subjected to hypotonic shock by incubation with 20 mM Tris-HCl (pH 7.5) for 2 min at room temperature to remove germ cells and to increase the purity of the Sertoli cell preparation. Thereafter, the hypotonic shock solution was removed and replaced with culture medium (lacking cytosine arabinoside) and cultured for another 4 days with daily medium exchanges prior to further experiments. The purity of the Sertoli culture was assessed by immunostaining for vimentin.

RNA of Sertoli cell cultures from individual mice was isolated using the RNeasy Mini Kit from Qiagen, according to the manufacturer’s protocol (Qiagen, Hilden, Germany). Each RNA preparation was subjected to RNase free DNase I (Qiagen) digestion to remove possible contamination of genomic DNA. The quantity and integrity of the isolated RNA was assessed with a NanoDrop® ND-1000 UV-Vis spectrophotometer (NanoDrop Technologies, Rockland, USA) and a quality profile with an Agilent 2100 bioanalyzer (Agilent Technologies, Waldbronn, Germany).

#### Microarray analysis of Sertoli cell mRNA from AMH-*Cre*/Wt and Wt/Wt mice

To reduce mistakes through differences in AMH-*Cre* expression in individual animals, Sertoli cell cultures of 6 heterozygous mice were collected. Due to the high similarity between individual wild type mice, only 3 animals were used for the RNA sample preparation of wild type mice. The same amount of total RNA of each animal was pooled together. Biotin-16-UTP labeling of the pooled probes (1 µg of RNA) was performed with the MessageAmp™ II-Biotin Enhanced Single Round aRNA Amplification Kit (Applied Biosystems, Darmstadt, Germany). The synthesized biotin-16-UTP-labeled cRNA was fragmented in the presence of Mg^2+^ and 10 µg of each sample preparation was loaded onto CodeLink™ Mouse Whole Genome Bioarray slides (2 arrays per sample, 300033-6PK, Applied Microarrays, Tempe AZ, USA). Hybridization was done at 37°C/300 rpm for 18 h in a Minitron shaker incubator (Infors AG, Bottmingen, Germany). Thereafter, the microarrays were washed and incubated with Cy-5-coupled streptavidine according to the manufacturer’s protocol (Amersham Biosciences, Freiburg) to visualize hybridized complexes. The labeled arrays were scanned using a GenePix 4000 B scanner and GenePix Pro 4.0 software (Axon Instruments, Arlington, USA). Scanned Images were analyzed using the CodeLink Expression Analysis software. The expression values were normalized by quantile normalization [34] and log2 transformed. Technical replicates were averaged and fold changes were calculated. The resulting gene list was subjected to the Database for Annotation, Visualization and Integrated Discovery (DAVID) [35] for annotation and overrepresentation analysis of gene categories. Gene lists were analyzed with Ingenuity Pathway Analysis (Ingenuity® Systems, Redwood City, USA) to detect altered canonical pathways. The microarray data was submitted to the Gene Expression Omnibus (GEO) database at the NCBI/NIH (http://www.nbci.nlm.nih.gov/projects/geo) and the accession numbers from GEO are as follows: GSM700341, GSM700342, GSM700343 and GSM700344. All data are MIAME compliant.

### Protein Analyses

#### Isolation of enriched organelle and cytosolic fractions from testes for analyses of peroxisomal and antioxidant proteins

Testes were homogenized with a Potter-Elvehjem homogenizer (Braun, Melsungen, Germany) in homogenization medium (5 mM MOPS, pH 7.4, 250 mM sucrose, 1 mM EDTA, 0.1% [v/v] ethanol, 0.2 mM dithiothreitol, 1 mM 6-aminocapronic acid, supplemented with 10% protease inhibitor mix M from SERVA, Heidelberg, Germany) for 2 min with a single stroke at 1,000 rpm. The homogenate was centrifuged at 500 g for 10 min to remove cell debris and nuclei. The supernatant was collected and re-centrifuged at 120,000 g at 4°C for 30 min to yield the mixed organelle pellet and the cytosolic supernatant, which were stored at −80°C.

#### Isolation of nuclear extracts from testes for transcription factor analyses

Nuclear extracts from testes were isolated with the ProteoJET™ Cytoplasmic & Nuclear Protein Extraction Kit according to the manufacturer’s protocol (Fermentas GmbH, St. Leon-Rot, Germany). Briefly, testes were treated with the cell lysis buffer and the lysate was centrifuged at 500 g to yield the nuclear fraction. The supernatant was further centrifuged at 20,000 g for 15 min at 4°C to remove most organelles and yield an enriched cytoplasmic fraction. The nuclear pellet was washed with the Nuclei Wash Buffer provided in the kit and lysed in the Nuclei Lysis Reagent by shaking for 15 min at 4°C with 1,200 rpm. Nuclear lysates were centrifuged at 20,000 g for 5 min at 4°C to obtain clear nuclear extracts.

#### Western blot analyses

Protein concentrations were determined by the Bradford protein assay (Bio-Rad, Munich, Germany) using BSA as standard. Protein samples (20 µg) were separated on 12% SDS-PAGE and further processed for Western blot analyses with the alkaline phosphatase-induced chemiluminescence technique as described by Nenicu and colleagues [28]. Primary and secondary antibodies are listed in Tables 2 and 3. The blots were exposured to Kodak Biomax MR Films. Bands on films were quantified with a Bio-Rad Gel Doc 2000 system. All Western blot analyses were performed three times and represent data from three individual experiments. An example for the specificities of the antibodies and method used is presented in Figure S2 (complete ABCD3 immunoblot).

**Table 2:** List of primary antibodies.

Antigens	Species antibodies raised in	Dilution(IF)	Dilution(WB)	Supplier
ATP-binding cassette transporter, sub-family D,member 3, peroxisomal membrane protein, mouse	Rabbit, polyclonal	1∶100	1∶100	Gift from Alfred Völkl, University of Heidelberg, Germany
p53 binding protein 1 (53BP1), rabbit	Rabbit, polyclonal	1∶200	–	Abcam plc, Cambridge, UK, Cat. No: ab21083
Catalase (CAT), mouse	Rabbit, polyclonal	1∶2,000	1∶10,000	Gift from Denis I. Crane, School of Biomol. Biophys. Sci., Griffith Univ., Nathan, Brisbane, Australia
Cleaved caspase-3, human	Rabbit, monoclonal	–	1∶1,000	New England Biolabs GmbH, Frankfurt am Main, Germany, Cat. No: 9664
Cre recombinase, P1 bacteriophage	Rabbit, polyclonal	–	1∶1,000	Covance, HISS Diagnostics GmbH, Freiburg, Germany, Cat. No: RPB-106C
Cyclooxygenase 2 (COX2), mouse	Rabbit, polyclonal	–	1∶1,000	Enzo Life Sciences GmbH, Loerrach, Germany, Cat. No: ALX-210-711
Glyceraldehyde 3-phosphate dehydrogenase (GAPDH), rabbit	Mouse, monoclonal	–	1∶10,000	HyTest Ltd, Turku, Finland, Cat. No: 5G4
Heme oxygenase-1 (HMOX1), rat	Rabbit, polyclonal	–	1∶2,000	Assay Designs, Inc. Michigan, USA, Cat. No: SPA-895
Histone H3, human	Rabbit, polyclonal	–	1∶1,000	New England Biolabs GmbH, Frankfurt am Main, Germany, Cat. No: 9715
4-Hydroxynonenal (4-HNE), keyhole limpet hemocyanin	Mouse, monoclonal	–	1∶100	Cosmo Bio Co., LTD., Tokyo, Japan, Cat. No: MHN-020P
3-Ketoacyl-CoA thiolase B (Thiolase), mouse	Rabbit, polyclonal	–	1∶6,000	Gift from Paul P. von Veldhoven, Catholic University Leuven, Belgium
Nuclear factor erythroid 2-Related Factor (NRF2), human	Rabbit, polyclonal	1∶300	1∶400	Santa Cruz Biotechnology Inc., Heidelberg, Germany, Cat. No: sc-13032
Nuclear factor κB (NF-κB) p65, human	Rabbit, polyclonal	–	1∶1,000	New England Biolabs GmbH, Frankfurt am Main, Germany, Cat. No: 3034
Peroxisomal biogenesis factor 5 (PEX5), mouse	Mouse, monoclonal	–	1∶200	BD Transduction Laboratories, USA. Cat. No: 611594
Peroxisomal biogenesis factor 13 (PEX13), mouse	Rabbit, polyclonal	1∶2,000	1∶6,000	Gift from Denis I. Crane (address see above)
Peroxisomal biogenesis factor 14 (PEX14), mouse	Rabbit, polyclonal	1∶4,000	1∶20,000	Gift from Denis I. Crane (address see above)
Phospho-c-Jun (Ser63) II, human	Rabbit, polyclonal	–	1∶800	New England Biolabs GmbH, Frankfurt am Main, Germany, Cat. No: 9261S
Phospho-NF-κB p65 (Ser536), human	Rabbit, monoclonal	–	1∶1,000	New England Biolabs GmbH, Frankfurt am Main, Germany, Cat. No: 3033
Phospho-p38 MAP Kinase (Thr180/Tyr182),human	Rabbit, polyclonal	–	1∶1,000	New England Biolabs GmbH, Frankfurt am Main, Germany, Cat. No: 9211S
Phospho-p44/42 MAPK (Erk1/2), human	Mouse, monoclonal	-	1∶1,000	New England Biolabs GmbH, Frankfurt am Main, Germany, Cat. No: 9106S
Phospho-SAPK/JNK (Thr183/Tyr185), human	Rabbit, polyclonal	–	1∶1,000	New England Biolabs GmbH, Frankfurt am Main, Germany, Cat. No: 9251S
p38 MAP Kinase, human	Rabbit, polyclonal	–	1∶1,000	New England Biolabs GmbH, Frankfurt am Main, Germany, Cat. No: 9212
Stress-activated protein kinase/Junamino-terminal kinase SAPK/JNK, human	Rabbit, polyclonal	–	1∶1,000	New England Biolabs GmbH, Frankfurt am Main, Germany, Cat. No: 9252
Superoxide dismutase 2 (SOD2), rat	Rabbit, polyclonal	1∶5,000	1∶6,000	Research Diagnostics, Inc., NJ, USA, Cat. No: RDI-RTSODMabR
α–tubulin, mouse	Mouse, monoclonal	–	1∶5,000	Sigma, Steinheim, Germany. Cat. No: T5168
Vimentin clone VIM 13.2, human	Mouse, monoclonal	1∶1,000	–	Sigma, Steinheim, Germany. Cat. No: V5255

**Table 3:** List of secondary antibodies and counterstaining of nuclei.

Secondary detection system used	Host	Method	Dilution	Supplier
anti-Mouse-IgG (whole molecule)-alkaline phosphatase	Goat	WB	1∶20,000	Sigma, Steinheim, Germany. Cat. No: A3562
anti-Rabbit-IgG (whole molecule)-alkaline phosphatase	Goat	WB	1∶20,000	Sigma, Steinheim, Germany. Cat. No: A3687
anti-Rabbit-IgG AlexaFluor488	Donkey	IF	1∶500	Molecular Probes/Invitrogen, Cat. No: A21206
anti-Mouse-IgG Texas Red	Horse	IF	1∶1,000	Vertor laboratories, Inc, Burlingame, USA, Cat. No: TI-2000
Hoechst 33342 (1 µg/ml) nucleic acid staining	–	IF	1∶750	Molecular Probes/Invitrogen, Carlsbad, CA, USA, Cat. No: A11007
TOTO-3 iodide	–	IF	1∶750	Molecular Probes/Invitrogen, Carlsbad, CA, USA, Cat. No: T3604

#### Immunofluorescence analyses

Testes were removed from three individual mice for every genotype and fixed by immersion with 4% depolymerized paraformaldehyde (PFA), containing 2% sucrose in phosphate-buffered saline (PBS) (150 mM NaCl, 13.1 mM K_2_HPO_4_, 5 mM KH_2_PO_4_, pH 7.4) at 4°C overnight. The fixed testes samples were embedded into paraffin (Paraplast, Sigma, St. Louis, MO, USA) using a Leica TP 1020 automated vacuum infiltration tissue processor. Paraffin sections (4 µm) were cut with a Leica RM2135 rotation microtome. The detailed protocol for embedding procedures and subsequent immunofluorescence analyses was described previously from our group [28]. Dilutions of the primary and secondary antibodies used are listed in Tables 2 and 3. Negative controls were processed in parallel by addition of TBST buffer instead of the first antibodies. Nuclei were visualized with 1 µM TOTO-3 iodide for 10 min at room temperature (Table 3). Samples were analyzed by confocal laser scanning microscopy (CLSM) with a Leica TCS SP2 (Leica Mikrosysteme Vertrieb GmbH, Wetzlar, Germany). Examples of negative control sections without primary antibody are shown in Figure S3.

### TUNEL Test for Apoptosis Detection

Apoptotic cells on paraffin sections were detected using the ApopTag® Fluorescein In Situ Apoptosis Detection Kit (S7110, Millipore GmbH, Schwalbach, Germany) by direct TdT-mediated dUTP-biotin nick end labeling (TUNEL), according to the manufacturer’s protocol. Testes from three animals were analyzed for each genotype. For quantification of apoptosis rates in each animal, TUNEL-positive cells in 300 cross-sections of seminiferous tubules, obtained from 8 sections of different testis regions were counted.

### Statistical Analysis for Real-time PCRs and the Morphometric Measurements

The significance value of homogeneity of variances was 0.006 (less than 0.05), wherefore the differences between the relative *Cre* transgene allelic abundances of real-time PCR results on genomic DNA were analyzed with the Kruskal-Wallis test using SPSS software.

The alterations of the apoptotic cell ratio between distinct genotypes were analyzed with the ANOVA test using SPSS software, because the significance value of homogeneity of variances was 0.830 (more than 0.05). The significance of differences between all genotypes was further analyzed with the t-test.

**Figure 2:**
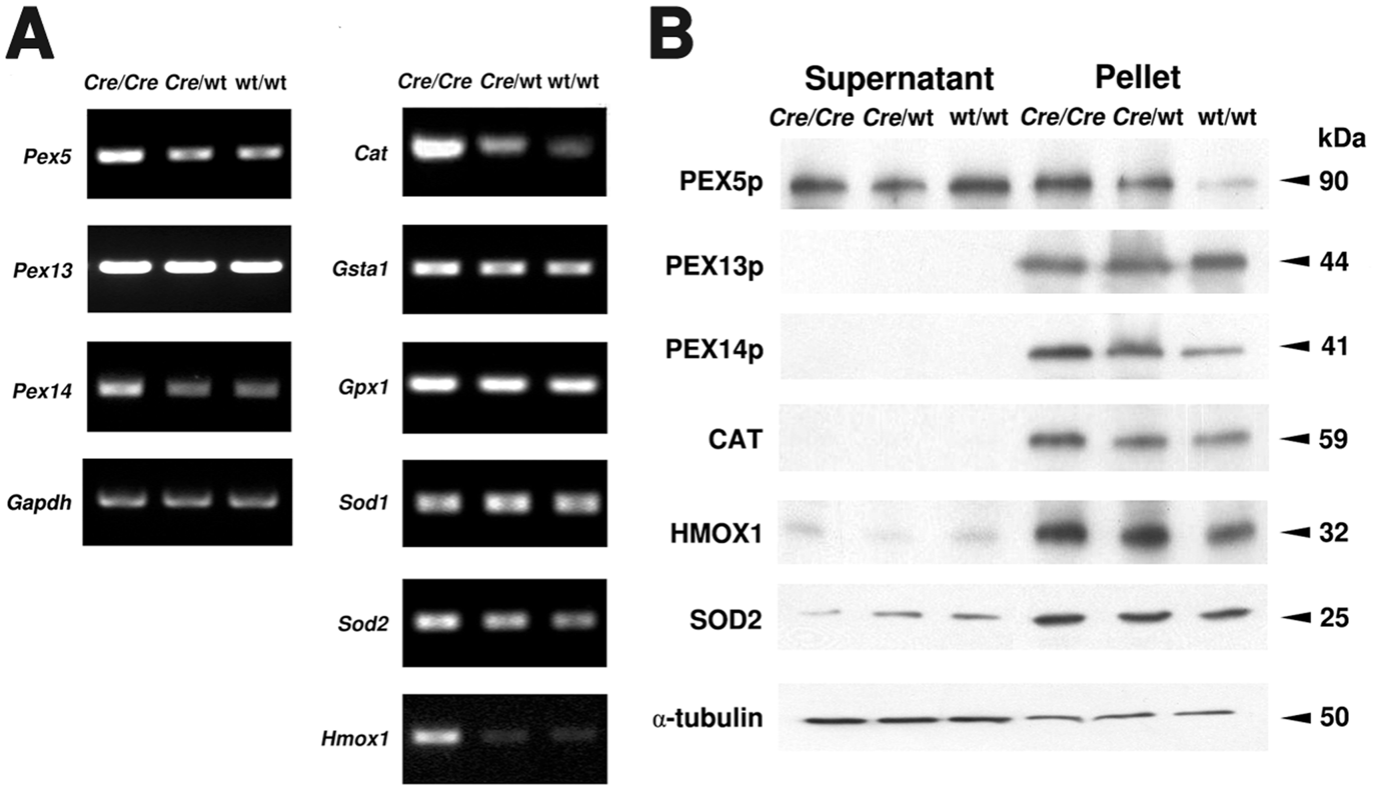
Peroxisome biogenesis and ROS metabolism are affected by *Cre* recombinase expression. **A**) Semiquantitative RT-PCR analyses for the genes involved in peroxisome biogenesis (*Pex5*, *Pex13*, *Pex14*) and ROS metabolism (*Cat*, *Gsta1*, *Gpx1*, *Sod1*, *Sod2*, *Hmox1*) on cDNAs prepared from total testicular RNA (genotypes are indicated), *Gapdh* as control. For abbreviation of gene names see Table 1. **B**) Western blot analyses of peroxisome biogenesis (PEX5p, PEX13p, PEX14p) and ROS metabolic (CAT, HMOX1, SOD2) proteins in cytosolic fractions (supernatant) and enriched organelles (pellet) of total testis samples (genotypes and protein masses are indicated). The abundance of α-tubulin was used as a loading control. 20 µg of protein were loaded per lane.

**Figure 3:**
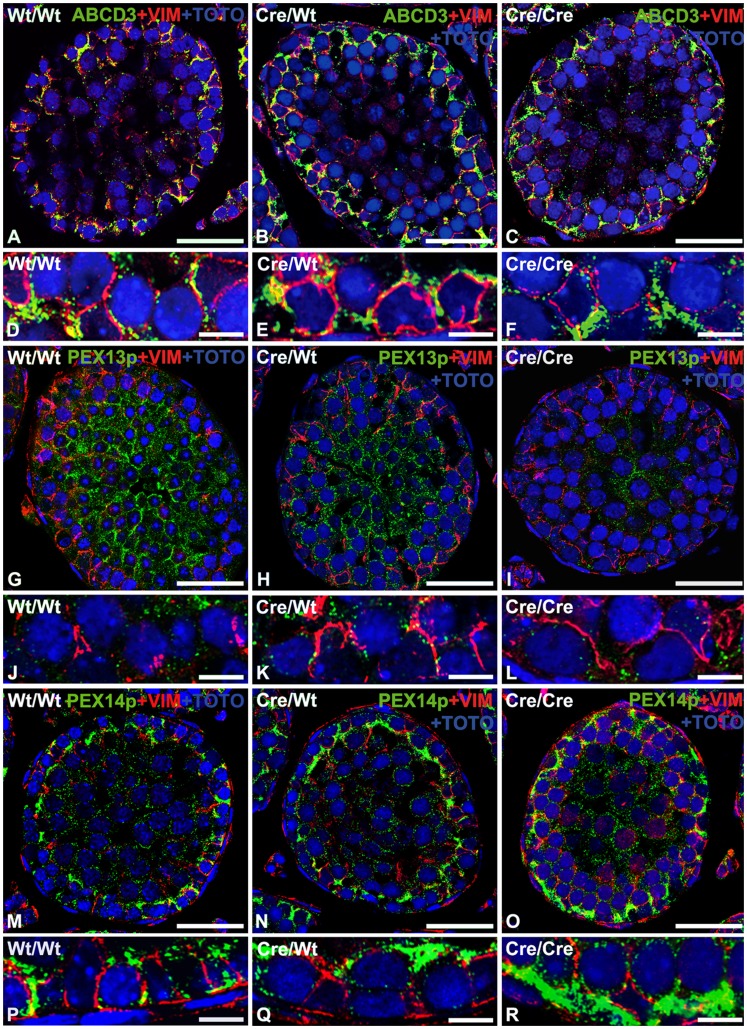
Double-immunofluorescence analysis reveals that AMH-*Cre*-expression induces peroxisome proliferation and membrane protein alterations. Double immunofluorescence for peroxisomal membrane proteins (green) and the intermediate filament protein vimentin as marker for Sertoli cells (red) with counterstaining of nuclei with TOTO-3 iodide on paraffin sections from the testis of wild type (A, D, G, J, M, P), heterozygous AMH-*Cre*-transgenic mice (B, E, H, K, N, Q) and homozygous AMH-*Cre*-transgenic mice (C, F, I, L, O, R). Peroxisomes are proliferated and the lipid transporter ABCD3 is increased in individual peroxisomes in Sertoli cells in the testis of AMH-*Cre*-transgenic animals in comparison to wild type animals (A–F). A similar pattern is observed for PEX14p, a peroxisomal biogenesis protein with highest abundance in Sertoli cells, which was strongly induced in AMH-*Cre*-transgenic mice (M–R). In contrast, PEX13p, a peroxisomal biogenesis protein with highest abundance in germ cell peroxisomes is not increased in Sertoli cells and rather decreased in the germ cell population (G–L). Size of bars is: A, B, C, G, H, I, M, N, O: 25 µm; D, E, F, J, K, L, P, Q, R: 10 µm.

## Results

Our group is investigating the effects of tissue-specific peroxisomal deficiency and we noted several unexplainable alterations of peroxisomal gene expression in the heterozygous animals with *Cre*-mediated PEX gene deletion. These alterations led us to reflect whether the sole expression of *Cre*-recombinase in a wild type background would affect the peroxisomal compartment and induce oxidative and metabolic stress reactions. We have therefore analyzed the effects of heterologous expression of *Cre-*recombinase in the testis of mice expressing the *Cre*-recombinase under control of the AMH promoter in Sertoli cells and used heterozygous and homozygous AMH-*Cre* animals in comparison to wild type mice in C57BL/6J genetic background to induce expression level-dependent alterations in Sertoli cells.

**Figure 4:**
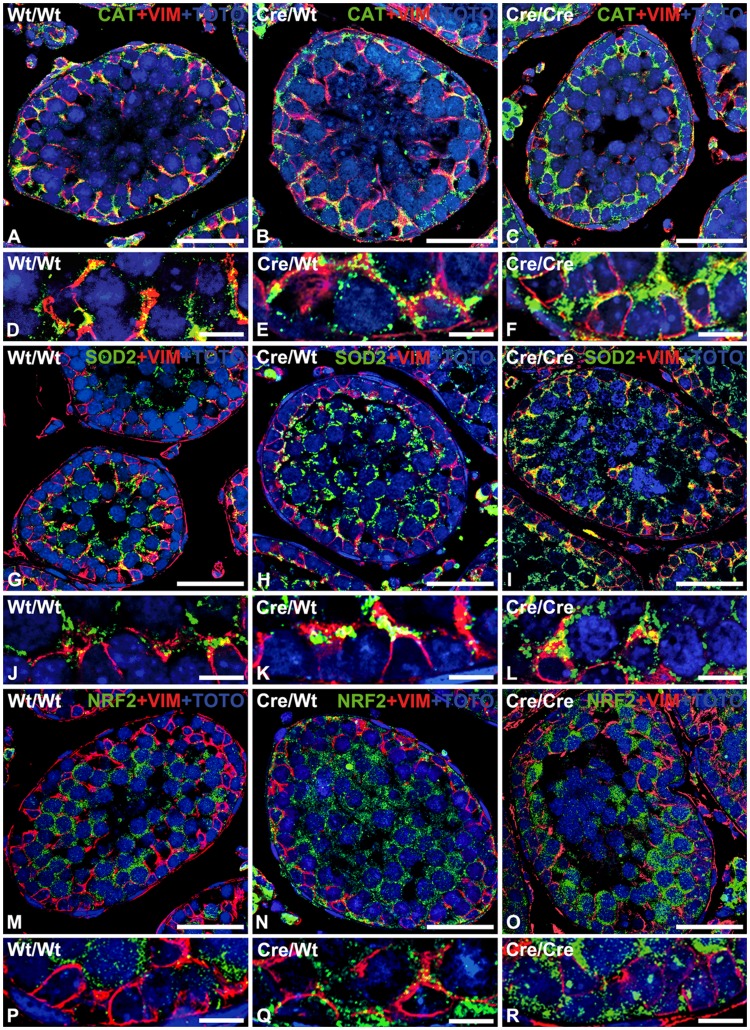
Immunofluorescence analysis reveals an increased antioxidative response in AMH-*Cre* expression Sertoli cells. Double immunofluorescence for peroxisomal catalase (CAT) (A–F), mitochondrial superoxide dismutase 2 (SOD2) (G–L) and the redox-sensitive transcription factor NRF2 (M–R) with the intermediate filament protein vimentin as marker for Sertoli cells (red) and counterstaining of nuclei with TOTO-3 iodide on paraffin sections from the testis of wild type (A, D, G, J, M, P), heterozygous AMH-*Cre*-transgenic mice (B, E, H, K, N, Q) and homozygous AMH-*Cre*-transgenic mice (C, F, I, L, O, R). Whereas catalase is mainly present in Sertoli cells, SOD2 and NRF2 show highest abundance in spermatocytes of wild type animals. Note that all three proteins were induced in Sertoli cells of AMH-*Cre*-transgenic mice (see higher magnification with regions of Sertoli cells). Size of bars is: A, B, C, G, H, I, M, N, O: 25 µm; D, E, F, J, K, L, P, Q, R: 10 µm.

**Figure 5:**
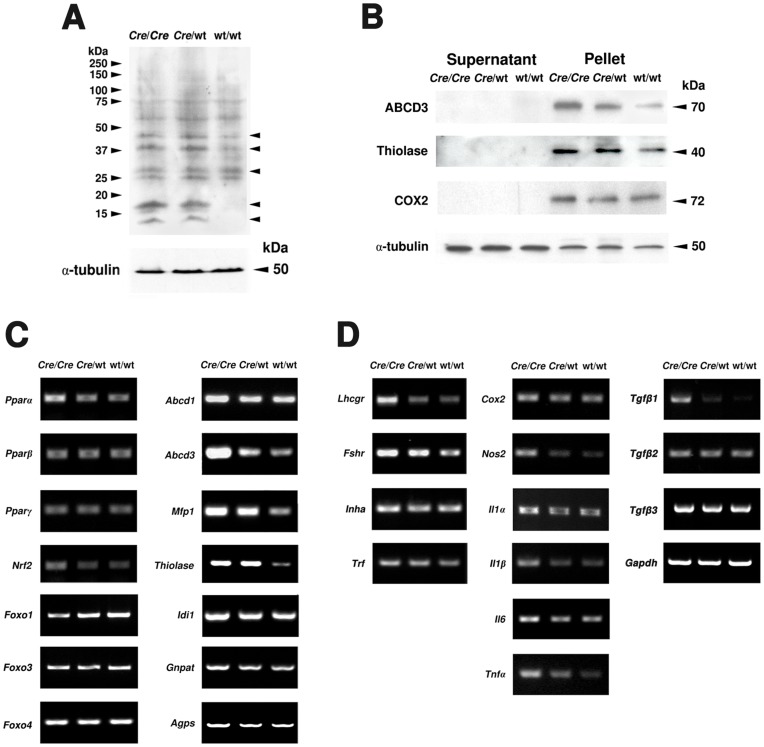
Alterations of gonadotropin receptors, paracrine regulators, proinflammatory genes, PPARs as well as lipid peroxidation. **A**) Western blot analysis of 4-HNE-modified lysine adducts in proteins of enriched organelle pellets of the testis (genotypes are indicated). The arrows on the right indicated the altered 4-HNE-modified proteins among the three genotypes. The abundance of α-tubulin was used as a loading control. 25 µg of protein were loaded per lane. **B**) Western blot analyses of proteins involved in peroxisomal lipid metabolism (ABCD3), the PPARα-inducible peroxisomal β-oxidation pathway 1 (Thiolase B) and COX2-protein in enriched organelle (pellet) and cytosolic fractions (supernatant) of the testis (genotypes and protein masses are indicated). The abundance of α-tubulin was used as a loading control. 20 µg of protein were loaded per lane. **C**) Semiquantitative RT-PCR analyses for PPARs (*Pparα*-, *Pparβ*-, *Pparγ*-mRNA), redox-dependent transcription factors (*Nrf2*-, *Foxo1*-, *3*-, *4*-mRNA) and genes involved in peroxisomal lipid metabolism (*Abcd1*-, *Abcd3*-mRNA), PPARα-induced peroxisomal β-oxidation pathway 1 (*Mfp1*, *Thiolase B*), peroxisomal cholesterol (*Idi1*) and ether lipid metabolism (*Gnpat*, *Agps*). The abundance of the *Gapdh* mRNA was used as a loading control. **D**) Semiquantitative RT-PCR analyses for expression of mRNAs for gonadotropin receptors (*Lhcgr*, *Fshr*), inhibin α (*Inha*) and genes involved in paracrine signaling (*Il1α*, *I1lβ*, *Il6*, *Tnfα*, *Tgfβ1*), as well as in proinflammatory pathways (*Cox2*, *Nos2*). The expression of the *Gapdh* mRNA was used as a loading control.

### *Cre* Expression in Sertoli Cells of the Testis Correlates with Allelic Abundance

Since also homozygous AMH-*Cre* animals were used for our experiments, genomic sequencing with a PCR-based method for walking in uncloned DNA was performed to unequivocally reveal that the insertion of the transgene into the mouse genome would not induce an undesired knockout of an endogenous gene by affecting exon regions. The sequencing results revealed that only a single copy of the transgene cassette, consisting of the human AMH-promoter (3,504 bp), fused to the *Cre*-gene (1,254 bp), followed by *MmMt1* (1,372 bp), was inserted into the large intron 3 (98,309 bp) of the *Plekha5* gene (ID: 109135) on chromosome 6 of the mouse genome (Fig. 1A).

To confirm the correct genotypes of the animals (AMH*-Cre*/AMH*-Cre*, AMH*-Cre*/Wt, and Wt/Wt) we have analyzed the allelic abundance (Fig. 1B) of the *Cre* transgene in the testes by quantitative genomic PCR. Homozygous AMH-*Cre*, heterozygous AMH-*Cre* and wild type animals without the *Cre*-transgene were easily identified by quantitative genomic PCR. The levels of mRNA expression (Fig. 1C) as well as the abundance of the *Cre* recombinase protein in the total testes samples (Fig. 1D) correlated well with the allelic abundance. In addition, RT-PCR analyses of microdissected samples of distinct testicular cell types revealed that the mRNA for *Cre* recombinase was only specifically expressed in Sertoli cells in a similar pattern already observed in total testis preparations, indicating that a comparison of the genotypes allows for an analysis of *Cre*-mediated effects in a gene dosage-dependent fashion in Sertoli cells (Fig. 1E).

**Figure 6:**
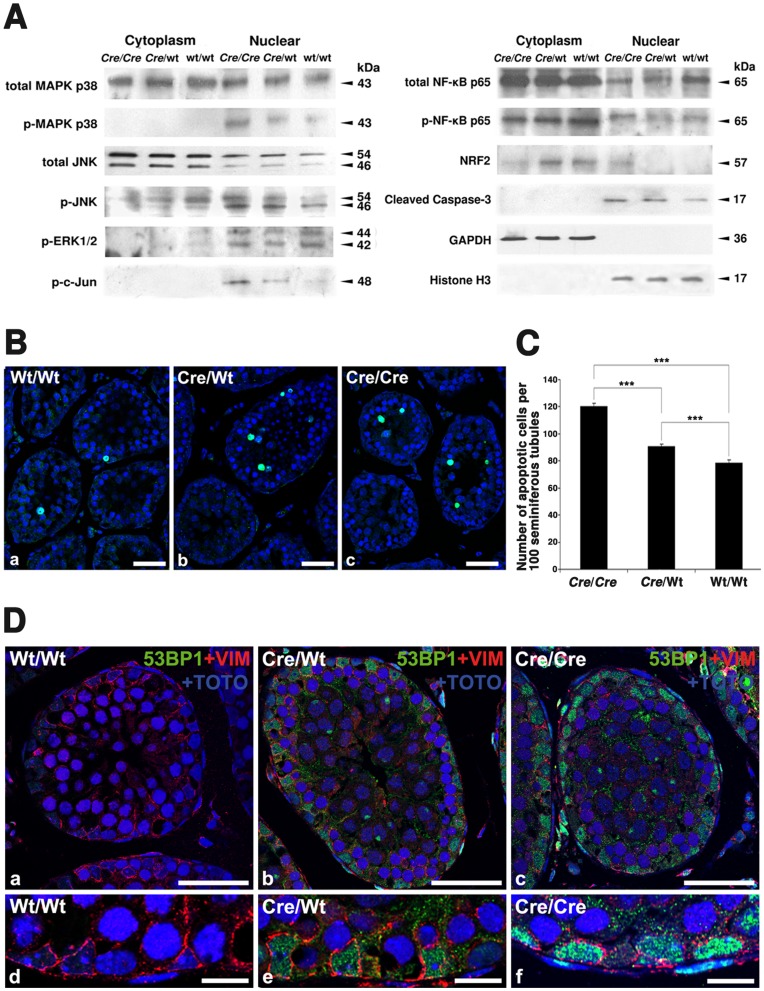
Signaling for antioxidative response and inflammation is activated and germ cell-apoptosis is increased in AMH-*Cre*-mice. **A**) Western blot analyses for signaling proteins of the MAP kinase family (p38, JNK, ERK1/2), c-Jun, NF-κB p65, NRF2 and caspase-3 in cytosolic and nuclear fractions of the testis (genotypes and protein masses are indicated). The abundances of GAPDH and Histone H3 were used as loading controls. 20 µg of protein were loaded per lane. **B**) TUNEL staining in seminiferous tubules of paraffin sections of the testis from wild type, heterozygous and homozygous AMH-*Cre* animals. Size of bars is 10 µm. **C**) Statistical analysis of TUNEL stainings revealed an increase of germ cell apoptosis in parallel to *Cre* allelic abundance (genotypes and number of apoptotic cells per 100 seminiferous tubules are indicated), *** p<0.001. **D**) Double immunofluorescence for the p53 binding protein 1 (green) and the intermediate filament protein vimentin as marker for Sertoli cells (red) with counterstaining of nuclei with TOTO-3 iodide on paraffin sections from the testis of wild type (a,d), heterozygous AMH-*Cre*-transgenic mice (b,e) and homozygous AMH-*Cre*-transgenic mice (c,f). The 53BP1 protein was induced in Sertoli cells of AMH-*Cre*-transgenic mice (see higher magnification with regions of Sertoli cells). Size of bars is: a-c: 25 µm; d-f: 10 µm.

### Peroxisome Biogenesis and ROS Metabolism are Affected by *Cre* Recombinase Expression

In order to assess alterations of the peroxisomal compartment, we have analyzed the expression of genes involved in peroxisome biogenesis, metabolism of reactive oxygen species (ROS) and peroxisomal lipid metabolism or of genes involved in the regulation of peroxisome-related gene expression and signal transduction pathways. From the 32 peroxin genes (according to the mouse nomenclature abbreviated as *Pex*), in the present study mainly the mRNA expression and protein abundance for *Pex5*/PEX5p, the shuttling receptor involved in the import of PTS1-containing matrix proteins into the organelle, and for *Pex13*/PEX13p as well as *Pex14*/PEX14p, two factors of the docking complex on the peroxisomal membrane for matrix protein import were analyzed. The *Pex5* and *Pex14* mRNA levels were increased already in total testes preparations of homozygous AMH*-Cre*/AMH*-Cre* animals compared to the wild type preparations (Fig. 2A) whereas the one for *Pex13* was not altered. Western blot analysis for abundance of PEX14p revealed a genotype-correlated increase with highest protein abundance in homozygous AMH-*Cre* animals. Also immunofluorescence analysis for this protein revealed its strong increase in Sertoli cells with concomitant induction of peroxisome proliferation in this cell type (Fig. 3M, N, O, P, Q, R). In contrast, PEX13p was most abundant in germ cells (Fig. 3G, H, I, J, K, L) and appeared slightly downregulated already in heterozygous *Cre*-transgenic mice (Fig. 3H, K) with homozygous animals exhibiting a stronger germ cell-specific downregulation (Fig. 3I, L). Comparable results for PEX13p were obtained also by Western blot analysis of total testis homogenates (Fig. 2B). Interestingly, PEX5p shifted from a primarily cytoplasmic localization in wild type to the organelle pellet in *Cre*-transgenic mice, suggesting the recruitment of this protein to the peroxisomal membrane.

**Figure 7:**
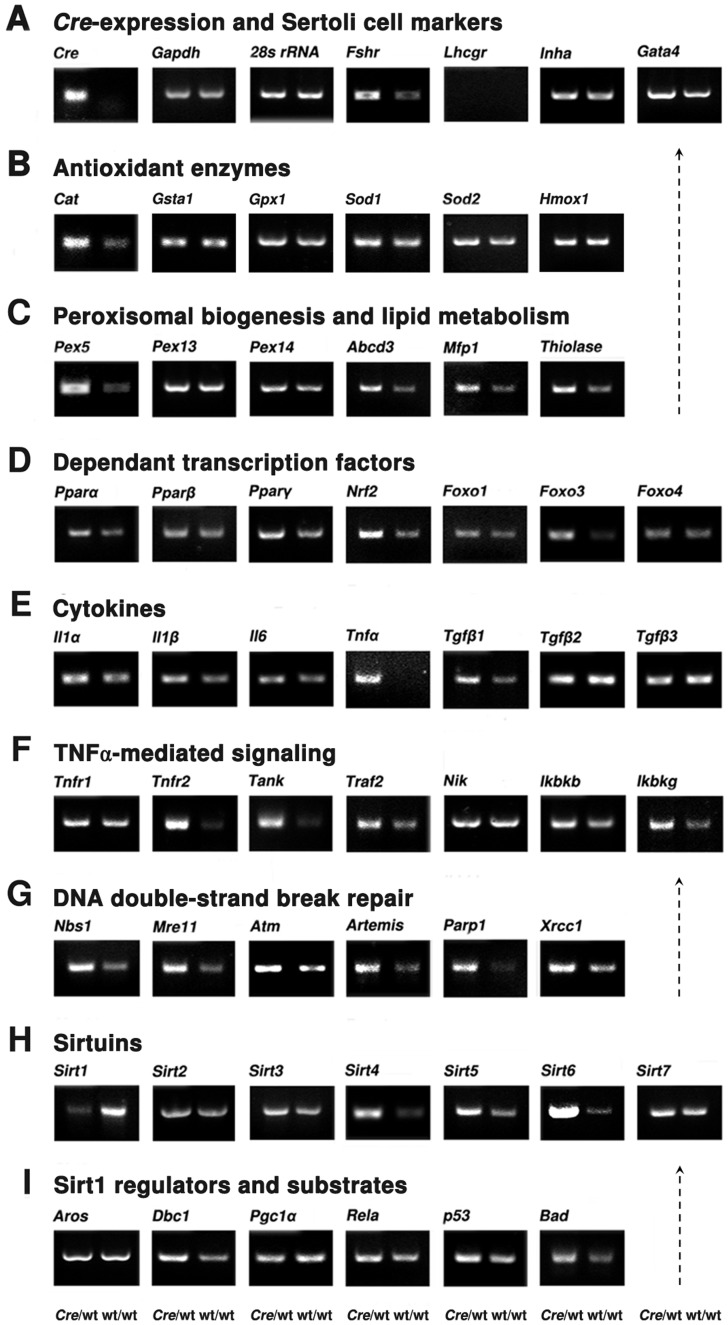
Differential gene expression in the testes and by microarray-analysis were observed in isolated Sertoli cells. Semiquantitative RT-PCR analyses for the RNA isolated from Sertoli cell cultures of AMH-*Cre*/Wt and Wt/Wt animals. **A**) *Cre*-transgene and gonadotropin receptors (*Lhcgr*, *Fshr*), transcription factor of Sertoli cells (*Gata4*), the expression levels of the *Gapdh* and *28s rRNA* mRNAs were used as control. **B**) Gene expression of antioxidant enzymes (*Cat*, *Gsta1*, *Gpx1*, *Sod1*, *Sod2*, *Hmox1*). **C**) Genes of proteins involved in peroxisomal biogenesis and lipid metabolism (*Pex5*, *Pex13*, *Pex14*, *Abcd3*, *Mfp1*, *Thiolase*). **D**) Analyses of *Ppar* mRNAs (*Pparα*, *Pparβ*, *Pparγ)* and the ones for redox-dependent transcription factor *(Nrf2*, *Foxo1*, *3*, *4*) **E**) Sertoli cell cytokine mRNAs (*Il1α*, *Il1β*, *Il6*, *Tnfα*, *Tgfβ1-3*). **F**) Expression levels of components involved in the TNFα-mediated signaling pathway (*Tnfr1*, *Tnfr2*, *Tank*, *Traf2*, *Nik*, *Ikbkb*, *Ikbkg*). **G**) Expression of mRNA for proteins involved in DNA double-strand break repair (*Nbs1*, *Mre11*, *Atm*, *Artemis*, *Parp1*, *Xrcc1*). **H**) Gene expression levels for all mammalian sirtuins (*Sirt1-7*). I) Analyses of mRNA expression levels for Activator (*Aros*) and inhibitor of *Sirt1* gene (*Dbc1*), as well as SIRT1 interacting partners and substrates (*Pgc1α, Rela, p53*, *Bad*).

Moreover, the mRNA expression for catalase, the most abundant antioxidative enzyme of the peroxisomal matrix, was significantly increased in total RNA preparations from testis already in heterozygous transgenic mice (Fig. 2A). Homozygous AMH-*Cre* animals showed a very strong increase of catalase mRNA expression. The results obtained on mRNA level could be corroborated for catalase on the protein level by Western blot analysis (Fig. 2B) as well as by immunofluorescence (Fig. 4A, B, C, D, E, F), indicating a strong increase of catalase in Sertoli cells (Fig. 4D, E, F). Similar effects were also noted on RNA and protein levels for antioxidative enzymes of other compartments, such as superoxide dismutase 2 (mitochondria) or heme oxygenase-1 (endoplasmic reticulum), whereas the mRNA levels for glutathion S-transferase 1 and glutathione peroxidase 1 were only slightly elevated (Fig. 2A and 2B). The mRNA for NRF2, a redox-dependent transcription factor regulating expression of most antioxidative enzymes including catalase, was slightly elevated in total RNA preparations from the testis (Fig. 5C). In contrast, the mRNA for the transcription factor FOXO1 was slightly decreased in homozygous AMH-*Cre* animals, whereas the ones for FOXO3 or FOXO4 were not significantly altered (Fig. 5C).

Immunofluorescence analysis revealed an increase in signal intensity for peroxisomal catalase and mitochondrial SOD2 mainly in Sertoli cells of the seminiferous tubules (Fig. 4A, B, C, D, E, F, G, H, I, J, K, L). Similarly NRF2 was also upregulated (Fig. 4M, N, O, P, Q, R), but only partly translocated to the nuclei of Sertoli cells (Fig. 4R). Furthermore, SOD2 and NRF2 were significantly increased in spermatocytes, suggesting the presence of oxidative stress also in germ cells (Fig. 4G, H, I, J, K, L, M, N, O, P, Q, R).

### Lipid Peroxidation Indicates Oxidative Stress in *Cre*-expressing Animals

As an indicator for oxidative stress and lipid peroxidation, we have analyzed the 4-hydroxynonenal (4-HNE)-modification of lysine residues in testes with distinct *Cre*-genotypes. The intensities of several 4-HNE-modified protein bands (Fig. 5A) were increased in organelle pellets from *Cre-*transgenic mice. Since 4-HNE metabolites and lipid peroxides are PPAR ligands and oxidized lipids are likely to be degraded in peroxisomes we have also analyzed the expression levels of mRNAs for PPARs and enzymes of peroxisomal β-oxidation. We found a significant upregulation of PPARα mRNA in the testis of homozygous AMH-*Cre* animals, whereas the mRNA expression levels for PPARβ and PPARγ in total RNA preparations from testis were not altered (Fig. 5C). In preparation from homozygous AMH-*Cre* testes, the strongest mRNA increase of genes encoding proteins involved in peroxisomal lipid metabolism was noted for the ABC-transporter ABCD3 (formerly PMP70), whereas the one for ABCD1 (formerly ALDP) showed only a minor increase. Furthermore, we found a strong induction of mRNAs encoding the multifunctional protein 1 (MFP1) and thiolase, two enzymes of the PPARα-induced peroxisomal β-oxidation pathway 1, already in heterozygous animals (Fig. 5C). In contrast, the mRNAs for enzymes of peroxisomal cholesterol (isopentenyl-diphosphate delta isomerase, IDI1) and ether lipid metabolism (glyceronephosphate O-acyltransferase, GNPAT and alkylglycerone phosphate synthase, AGPS) were only mildly induced. The alterations of mRNA expression levels of ABCD3 and thiolase were corroborated by corresponding changes in protein abundance in Western blot analyses of organelle fractions (Fig. 5B). Moreover, immunofluorescence analysis revealed the strongest upregulation of ABCD3 in Sertoli cells with only minor increases in germ and Leydig cells (Fig. 3A, B, C, D, E, F).

### Sertoli Cell-mediated Stress Induces Alterations in Expression of Gonadotropin Receptors, Paracrine Regulators and Proinflammatory Genes in AMH-*Cre*-transgenic Mice

As shown above, alterations of the abundance of the peroxisomal ABCD3 and the mitochondrial SOD2 proteins were observed also in germ and Leydig cells in AMH-*Cre* homozygous animals, indicating that paracrine factors might be released from Sertoli cells under oxidative stress conditions, influencing also other testicular cells types. We have therefore investigated proteins involved in endocrine and paracrine regulation of testis functions. The mRNAs for gonadotropin receptors were strongly upregulated in the testes of mice bearing the *Cre-*transgene (Fig. 5D). Interestingly, the *Lhcgr* was induced only in homozygous animals, whereas *Fshr* induction occurred already in heterozygous animals. Furthermore, mRNAs for proteins involved in paracrine signaling, such as IL1α, IL1β, IL6, TNFα and TGFβ1, were also increased, whereas the ones for TGFβ2 and 3 were not altered. In addition, the mRNAs of genes activated in proinflammatory conditions, like the *Cox2*- and *Nos2*-genes, were also upregulated in the testes of homozygous AMH-*Cre* mice (Fig. 5D). Similar to mRNA levels, the COX2 protein abundance was also elevated in homozygous AMH-*Cre* mice (Fig. 5B).

### Signal Transduction Pathways Involved in Apoptosis, Antioxidative Response and Inflammation are Activated in AMH-transgenic Mice

Already in H&E stained preparations an increase of altered germ cells, reminiscent for apoptosis were observed, wherefore we have analyzed the abundance and phosphorylation state of different MAP kinase family signal transduction molecules, NF-κB and activated caspase-3. In total testis preparations, the overall abundance of MAPK p38 was not significantly altered in *Cre-*transgenic- compared to wild type mice. The amount of the phosphorylated p38 (pMAPK p38), however, was increased in nuclear extracts from testes of *Cre*-transgenic mice (Fig. 6A). Similarly, the relative abundance of the activated form of JNK (mainly p46 JNK) was significantly increased in the nuclear extracts in a dose-dependent manner (Fig. 6A). Furthermore, the phosphorylated and activated form of c-Jun (p-c-Jun), downstream of JNK signaling, was increased in a similar fashion. In contrast, the abundance of phosphorylated ERK1/2, another member of the MAPK-family, was not altered in *Cre*-transgenic mice compared to the wild type animals (Fig. 6A).

As expected, NF-κB p65 was primarily localized in the cytosolic fractions and only a minor amount was present in the nuclear extracts. The ratio of phosphorylated (p-NF-κB p65) to unphosphorylated NF-κB p65, however, was increased in the nuclear fractions of *Cre*-transgenic mice, suggesting an activation of the NF-κB pathway (Fig. 6A). NRF2, another ROS-activated signal transduction molecule was primarily localized in the cytosolic fraction in wild type mice. In homozygous *Cre*-transgenic mice, we observed a significant shift of NRF2 into the nuclear fraction, indicating its activation in these animals (Fig. 6A). Since signaling molecules of three distinct pathways (MAPK p38, JNK and NF-κB), implicated in the regulation of cell survival, proliferation and apoptosis [36], [37], were activated in *Cre*-transgenic animals, we also analyzed the abundance of the cleaved form of caspase-3. Indeed, we found that the amount of the activated caspase was increased in nuclear extracts of testicular cells in AMH-*Cre-*transgenic animals in a dose-dependent manner (Fig. 6A), wherefore we analyzed the apoptosis rates in the seminiferous tubules of AMH*-Cre*/AMH-*Cre*, AMH-*Cre*/Wt, and Wt/Wt mice by TUNEL staining (Fig. 6B). Quantification of TUNEL positive cells revealed a moderate but significant increase in number of apoptotic germ cells already in heterozygous in comparison to wild type testes (p<0.001) and a more pronounced increase in homozygous *Cre*-transgenic testes (p<0.001) (Fig. 6C). No apoptosis of Sertoli cells was noted, suggesting a higher capacity of this cell type to counteract oxidative and cytokine-mediated stress as well as *Cre*-induced DNA damage [7], [8]. One of the compensatory mechanisms could be a higher capacity of Sertoli cells of transgenic AMH-*Cre* mice for DNA double-strand break repair, wherefore we have also analyzed the recruitment of the p53 binding protein 1 (53BP1) by immunofluorescence analysis [38]. Indeed, an increased staining for the 53BP1 protein was observed already in Sertoli cell nuclei of heterozygous AMH-*Cre* mice, which was even more pronounced in homozygous AMH-*Cre* animals (Fig. 6D).

### Effects Observed on the Molecular Level in Total Testis Preparations could also be Verified in RNA Preparations of Primary Sertoli Cell Cultures of Heterozygous AMH-*Cre* Animals

Generally heterozygous *Cre* mice are used for the generation of a “gene of interest (GOI)”-knockout by the *Cre*-*loxP* technology (e.g. promoter X-*Cre*/Wt/GOI-*loxP*_ΔY/Wt) in matings with mice homozygous for the floxed GOI. To analyze whether all observed gene alterations from total testis RNA preparations were specific for Sertoli cells, we have used primary Sertoli cell cultures from AMH-*Cre*/Wt mice and Wt/Wt mice for RT-PCR analyses. Indeed, a comparable expression pattern was observed as in total testis preparations for genes of peroxisomal and mitochondrial proteins (Fig. 7B and 7C) and corresponding transcription factors (Fig. 7D). The mRNAs for FSH receptor (Fig. 7A) and Sertoli cell cytokines (Fig. 7E) were upregulated, with the *Tnfα* mRNA showing the highest increase also in Sertoli cells as compared to total testis preparations. Interestingly, in addition to the increase of *Pparα* mRNA expression level, clear inductions of *Pparγ*-, *Nrf2*- and *Foxo3*-mRNAs were observed in the heterozygous *Cre* expressing Sertoli cells (Fig. 7D). Moreover, the *Gata4* mRNA (Fig. 7A), encoding a transcription factor of Sertoli cells that is increased in many testicular diseases in patients, was also clearly upregulated in Sertoli cells with heterozygous *Cre* expression.

Since with primary Sertoli cells we have obtained similar results as with complete testis preparations, we have done a microarray analysis with CodeLink™ Mouse Whole Genome Bioarray slides to check for broad alterations in gene expression. Since the fold changes of genes were only in a range of 5.043 (highest upregulated gene: *Agapat6*) and −4.825 (lowest downregulated gene: *S100a9*), we used a cut-off ratio of fold change (FC) >1.5 to perform the Ingenuity Pathway Analysis. With a cut-off ratio of 3 or 2 only a limited amount of genes were listed in each canonical pathway, suggesting that these calculations would not be biologically relevant. The top 10 up- and downregulated genes and the top 10 altered canonical pathways are listed in Table S1. Interestingly, the analysis of processed data with the Ingenuity Pathway Analysis (IPA®) software revealed that the TNFR2 pathway was listed within the top 10 ranking of pathways with highest significant changes (with a -log (p-value) of 1,83E00 and ratio of 2,26E-01), whereas the TNFR1-pathway was only ranked on place 61 (see Table S2). The pathways for IL-1-, and TGFβ-signal transduction were listed at ranks 54 and 88. In contrast to those pathways, within the top 10 ranking also the “role of JAK family kinases in IL-6-type cytokine signaling” and “interferon signaling” were listed as well as an “antigen presentation pathway”, “activation of IRF by cytosolic pattern recognition receptors (PRR)” and the “role of PRR in the recognition of bacteria and viruses”, suggesting a possible reaction against the “virus-derived” RNA of *Cre* recombinase. In addition, “calcium-induced apoptosis”, “PKCθ signaling” and “GNRH signaling” were in the top 10 ranking. Since we have found the highest alterations of *Tnfα* mRNA level in the Sertoli cell by RT-PCR experiments, we decided to verify the microarray results of the TNF-receptor pathways first. Indeed, the mRNA level for TNFR1 was only slightly altered, whereas the expression levels for most components of the TNFR2-dependent signal transduction pathway were induced (Fig. 7F).

### DNA Repair Proteins and Sirtuin Deacetylases are Affected in Sertoli Cells

Since *Cre* recombinase induced DNA damage at pseudo-*loxP* sites in the genome will affect both DNA strands [7], [8] and we have found a strong recruitment of 53BP1 in Sertoli cell nuclei of transgenic mice (Fig. 6D), the mRNA levels for proteins involved in DNA double-strand break repair processes (Fig. 7G) were also analyzed. Indeed, the expression of mRNAs for all of the DNA repair proteins were increased, including the one for ATM, a key regulator inducing p53 and mediating sirtuin deacetylase effects. Since alterations in nuclear sirtuins (SIRT1,6,7) could explain at least in part the effects of *Cre*-mediated DNA damage on antioxidant, proinflammatory and metabolic pathways, we have analyzed also the mRNA expression levels for all mammalian sirtuins (SIRT1-7) (Fig. 7H). Strong but opposite effects were noted on mRNA expressions for sirtuins involved in DNA repair processes (SIRT1,6), whereas the one for SIRT7 was only weakly upregulated. In addition, mitochondrial sirtuin (*SIRT3,4,5*) mRNAs were also increased in expression levels. Only *Sirt2* mRNA expression was minor affected. Moreover, the mRNA for the SIRT1 inhibitor DBC1 was increased and the ones for SIRT1 interacting partners and substrates for deacetylation altered (*Pgc1α*-, *Rela*-, *p53*- and *Bad*-mRNAs) (Fig. 7I), suggesting that the downregulation of *Sirt1* could provide the direct link between *Cre*-mediated DNA damage and most observed effects on lipid metabolic, antioxidant and proinflammatory pathways in Sertoli cells. Since SIRT1 actions on signal transduction pathways are very complex and depend on the cell type and tissue [39], future studies have to clarify the exact mechanism of *Cre*-mediated stress induction leading to mild germ cell apoptosis already in heterozygous AMH-*Cre* animals.

## Discussion

The *Cre*-*loxP* system is widely used for the generation of animal models with cell- or tissue-specific gene inactivation [1], [6]. Heterologous expression of the *Cre-*recombinase, however, has been shown to excert side effects, including reduction of cell proliferation and cell viability as well as induction of chromosome rearrangements due to recombination using cryptic *loxP* sites [7], [8]. Since we have used a mouse model with expression of *Cre-*recombinase under control of the AMH-promoter [30] for analysis of generalized peroxisome defects in Sertoli cells, we have also used this model to analyze the effects of sole *Cre-*expression on the peroxisomal compartment and stress-related pathways [40] in Sertoli cells and on other cell types in the testis. To our surprise, we found a *Cre*-mediated induction of peroxisome proliferation in Sertoli cells as well as differential alterations in the expression of a number of genes involved in peroxisomal metabolic pathways in the testis. Upregulation of catalase - the most abundant antioxidative enzyme of peroxisomes - correlated with the expression level of the *Cre-*recombinase, suggesting an adaptation of the peroxisomal compartment to increased oxidative stress. In fact, we could show that lipid peroxidation as an indicator for oxidative stress was increased in the testes of *Cre-*expressing mice. The increase of other central enzymes in antioxidant defense mechanisms, such as HMOX1 in the endoplasmic reticulum and mitochondrial SOD2, reflect that the increased need for antioxidant capacity was not restricted to peroxisomal enzymes only. Upregulation of these antioxidative enzymes could be mediated by NRF2- or NF-κB signaling, redox-sensitive transcription factors [41], [42], which were both induced in the testis of *Cre*-expressing mice, with NRF2 being translocated to a specific region in the nuclei near the nucleolus of Sertoli cells.

To better understand the increase in numerical abundance of peroxisomes and the increased lipid peroxidation in the testis of *Cre*-expressing mice, we analyzed whether also expression of genes and proteins involved in the biogenesis of peroxisomes or in peroxisomal lipid metabolism were altered. We observed an induction of PEX14p and a concentration of PEX5p onto the peroxisomal surface. Both peroxins are involved in peroxisomal protein import for matrix proteins, such as catalase [21]. Peroxisome proliferation can be induced via PPARα [43], [44], [45] and indeed, *Pparα* was significantly induced in the testis of AMH-*Cre-*expressing animals. Among the endogenous ligands for PPARs are lipid peroxides or eicosanoids that are generated under oxidative stress and proinflammatory conditions [46], [47] leading also to the increase of 4-HNE-modified lysine adducts in proteins that were more abundant in AMH-*Cre-*expressing animals.

PPARα induction leads to the transcriptional activation of genes for peroxisomal β-oxidation enzymes [13], [48] and this pathway in turn is involved in control of the homeostasis of PPARα ligands by their degradation [16], [49]. We could show that the expressions of the mRNAs for multifunctional protein 1 and the 3-ketoacyl-CoA thiolase B, both enzymes of the PPARα-inducible peroxisomal β-oxidation pathway 1, were induced in the testes of *Cre-*expressing mice in addition to ABCD3, a transporter for peroxisomal β-oxidation substrates. In addition to being PPARα ligands, secreted eicosanoids such as prostaglandins or leukotrienes, as well as cytokines are well-known mediators of proinflammarory signaling that have also been implicated in Sertoli cell-mediated paracrine regulation in the testis [50], [51], [52]. Indeed, the mRNAs for proinflammatory cytokines (*Il1α-*, *Il1β*-, *Il6*- and *Tnfα*-mRNA) and *Nos2*-mRNA, encoding the inducible form of the nitric oxide synthases, were increased in the testes of AMH-*Cre* expressing mice. We also observed that the amount of apoptosis of germ cells correlated with an increase of *Cre-*expression. Since *Cre* was specifically expressed in Sertoli cells as shown in microdissection experiments, the induction of germ cell apoptosis must be an indirect effect that could be mediated by the increased release of cytokines and eicosanoids due to *Cre*-exerted stress in Sertoli cells. Indeed, increased TGFβ1 leads to the opening of the blood-testis-barrier [53] and germ cell apoptosis [54]. Furthermore, TNFα perturbs cell junctions between Sertoli and germ cells and leads to premature release of germ cells from the seminiferous epithelium [55]. Interestingly, stress in Sertoli cells is frequently accompanied with increased levels of *Gata4*- and *Fshr*-mRNA expression [56], [57], [58], [59], which were also observed in our study already in heterozygous *Cre* expressing Sertoli cells. Indeed, upregulated FSHR signaling could lead to enhanced cytokine production and release from stressed Sertoli cells via the PKA pathway [60], [61].

In addition to cytokines, also 4-HNE has been proposed to play a role in cell signal transduction, in a variety of pathways affecting cellular adhesion, the cell cycle and apoptosis [62], [63]. 4-HNE, an α, β-unsaturated hydroxyalkenal, which is produced by lipid peroxidation of n-6 PUFA, is a potent cytotoxic and genotoxic compound [64]. 4-HNE induced apoptosis in various cell lines was accompanied by activation of JNK and caspase-3 [65], [66]. Indeed, our results indicate that major signaling pathways triggered by oxidative stress (NF-κB, MAPK p38, JNK) are activated. Furthermore, a significant activation of c-Jun and caspase-3 was demonstrated in our study, suggesting that apoptotic germ cell death might indeed be induced by these pathways also in *Cre-*expressing animals.

Cell toxicity and effects on cell viability of *Cre* have been shown to be inherent to its recombinase activity [7]. *Cre-*expression has been shown to induce chromosomal rearrangements in genomes that do not contain original *loxP* sequences, leading to the discovery of cryptic *loxP* sites that were described in the yeast and the mammalian genome [67], [68]. As depicted in Figure S1, a schematic overview made with the IPA® software, *Cre*-recombination induces DNA double-strand breaks that should induce the recruitment of 53BP1 and of proteins of the related DNA damage repair pathways [69]. Indeed, the recruitment of 53BP1 and an induction in expression of the mRNAs for DNA repair proteins was observed in this study. Interestingly, DBS1, a sensor for double-strand breaks, is deacetylated by sirtuin 1 (SIRT1) and thereby activated to induce the downstream DNA repair pathways via the ATM protein [70], [71]. By this mechanism SIRT1, belonging to the nuclear sirtuins that are able to deacetylate histones to control chromatin dynamics and DNA repair, is recruited to damaged DNA regions [72]. In addition to SIRT1, SIRT6 is also bound to chromatin and is involved in double-strand break repair by regulating the C-terminal binding protein (CtBP) and DNA protein kinase [73], [74], [75]. A deletion of *Sirt6* has been shown to lead to genomic instability, chromosome breaks and -fusions, higher sensitivity to genotoxic agents, such a H_2_O_2_ and to severe metabolic defects and aging [69], [76], [77]. Alterations of sirtuin levels could indeed explain part of the observed phenotype in our study. Whereas *Sirt6* was increased, probably to promote genomic stability in *Cre* expressing cells, *Sirt1* was strongly decreased under chronic stress conditions in Sertoli cells, possibly leading to derepression of NF-κB-dependent signaling [78] and subsequently to cytokine release from Sertoli cells. Sertoli cell-released TNFα could stimulate the TNFR2 pathway by an autocrine mechanism, which is supported by the increased mRNA levels of most TNFR2 signaling components in our study.

Oxidized lipids are strong ligands for PPARγ, which can bind to the promoter of the *Sirt1* gene, repressing its transcriptional activity [79]. Interestingly, the SIRT1 protein in turn deacetylates besides PPARγ also other proteins involved in metabolic regulation, signal transduction and apoptosis (PPARα, PGC-1, p53, NF-κB, FOXO3, BAD) [39], [77], [80], [81]. Thus, a decrease of SIRT1 could disturb cellular homeostasis and promote aging [39], [77]. In addition to a decrease of *Sirt1* expression, an increase of the expression level of *Dbc1*, encoding an inhibitor of SIRT1 activity [82], [83], [84], was observed in Sertoli cells of heterozygous AMH-*Cre* mice. Furthermore, *Sirt1* mRNA abundance is also regulated post-transcriptionally by differential binding of the HuR protein to the 3′UTR [82], [85], [86], [87]. Under oxidative stress conditions HuR can be phosphorylated by MAPK p38 or the check point kinase Chk2, leading to dissociation of the HuR-*Sirt1-*mRNA-complex and its degradation [82], [88]. Interestingly, *Sirt1* deficient mouse embryonic fibroblasts are resistant to chronic and sublethal doses of oxidative stress [39], [89], suggesting that Sertoli cells might be protected against apoptosis at least partially by this mechanism as well as MAPK p38 activation [36].

MAPK p38 and c-Jun activation as well as peroxisomal alterations correlated well with *Cre-*expression levels. Similar dose-dependent correlations were found also for *Fshr*, *Tnfα* and *Tgfβ1* mRNA expression levels in the testis of *Cre* expressing mice. These findings suggest that high *Cre* levels that are obtained by promoters with strong activity will lead to more *Cre*-induced side effects. Indeed, several reports indicate that high levels of *Cre-*expression might induce pathological alterations in diverse organ systems in mice [8], [9], [11], [90], [91] and can even lead to cardiomyopathy [10], the molecular pathogenesis of which, however, has not yet been published.

### Conclusions

Our study indicates that the sole expression of *Cre-*recombinase in AMH-*Cre* mice can lead to alterations of genes involved in peroxisomal biogenesis, ROS- and lipid metabolism, stress induced signal transduction pathways, expression of gonadotropin receptors as well as proinflammatory cytokines and apoptosis. We hypothesize that *Cre*-induced stress is mediated at least in part by the increased DNA damage and transduced by sirtuins and the repression release of important key regulators of signal transduction pathways (Figure S1). Therefore, wild type controls containing a heterozygous *Cre* genotype matched to the knockout animals, have to be generated as control animals by separate breedings in parallel, to insure that the analyzed phenotypic effects are due to the deletion of the floxed alleles and not to *Cre*-induced side effects.

## Supporting Information

Figure S1 **Schematic overview of the model for regulation and transduction of AMH-** ***Cre*** **recombinase induced stress in Sertoli and germ cells.**
(TIF)

Figure S2 **Western blot of cytosolic and enriched organelle fractions of testes of AMH-** ***Cre*** **/AMH-** ***Cre*** **, AMH-** ***Cre*** **/Wt and Wt/Wt mice, probed with an antibody against the peroxisomal membrane lipid transporter ABCD3.** The blot shows the high specificity of the antibody, which is necessary to obtain an optimal and reliable staining for the localization of organelles in tissue sections.
(TIF)

Figure S3 **Negative control sections (without primary antibody) of the testes of Wt/Wt and AMH-** ***Cre*** **/AMH-** ***Cre*** **mice, processed in parallel to double-immunofluorescence preparations presented in the manuscript, depicting only very few unspecific binding sites of the secondary antibodies and revealing the high reliability of the immunofluorescence protocol used.**
(TIF)

Figure S4 **Hematoxylin and eosin (H&E) staining of testis sections of sexually mature adult animals (P120) with Wt/Wt (A, B), AMH-** ***Cre*** **/Wt (C, D) and AMH-** ***Cre*** **/AMH-** ***Cre*** **(E, F) genotypes.** **A, C, E:** lower magnification overviews (bar: 50 µm); **B, D, F:** corresponding partial higher magnifications of the same regions (bar: 50 µm). Note the higher amount of apoptotic germ cells in a semininiferous tubule (seminiferous epithelium at stage 9) of a heterozygous *Cre* animal. In homozygous *Cre* mice also a higher apoptosis rate was noted (in stage 9 tubules, data not shown) and a mosaic pattern was observed, with few degenerated seminiferous tubules exhibiting severely impaired spermatogenesis located besides normal appearing ones with regular spermatogenesis.
(TIF)

Sequence S1 **Assembled sequence obtained by PCR-based genome walking proving the insertion of the transgene in the intron 3 region of the** ***Plekha5*** **gene on chromosome 6.** Sequencing was started in both directions from the AMH-*Cre* transgene cassette to obtain flanking regions at both sides.
(DOC)

Table S1 **Top 10 ranking of gene and canonical pathway alterations in primary** ***Cre*** **-expressing Sertoli cells (AMH-** ***Cre*** **/Wt versus Wt/Wt) obtained by Ingenuity Pathway Analysis (IPA® software).**
(DOC)

Table S2 **List of Ingenuity Pathway Analysis (IPA® software) - alterations of canonical pathways.**
(XLS)
