# Supplementary figures and images for: Cre-Mediated Stress Affects Sirtuin Expression Levels, Peroxisome Biogenesis and Metabolism, Antioxidant and Proinflammatory Signaling Pathways
Source: PLoS One. 2012 Jul 19;7(7):e41097. doi: 10.1371/journal.pone.0041097 (PMC3400606; doi:10.1371/journal.pone.0041097)

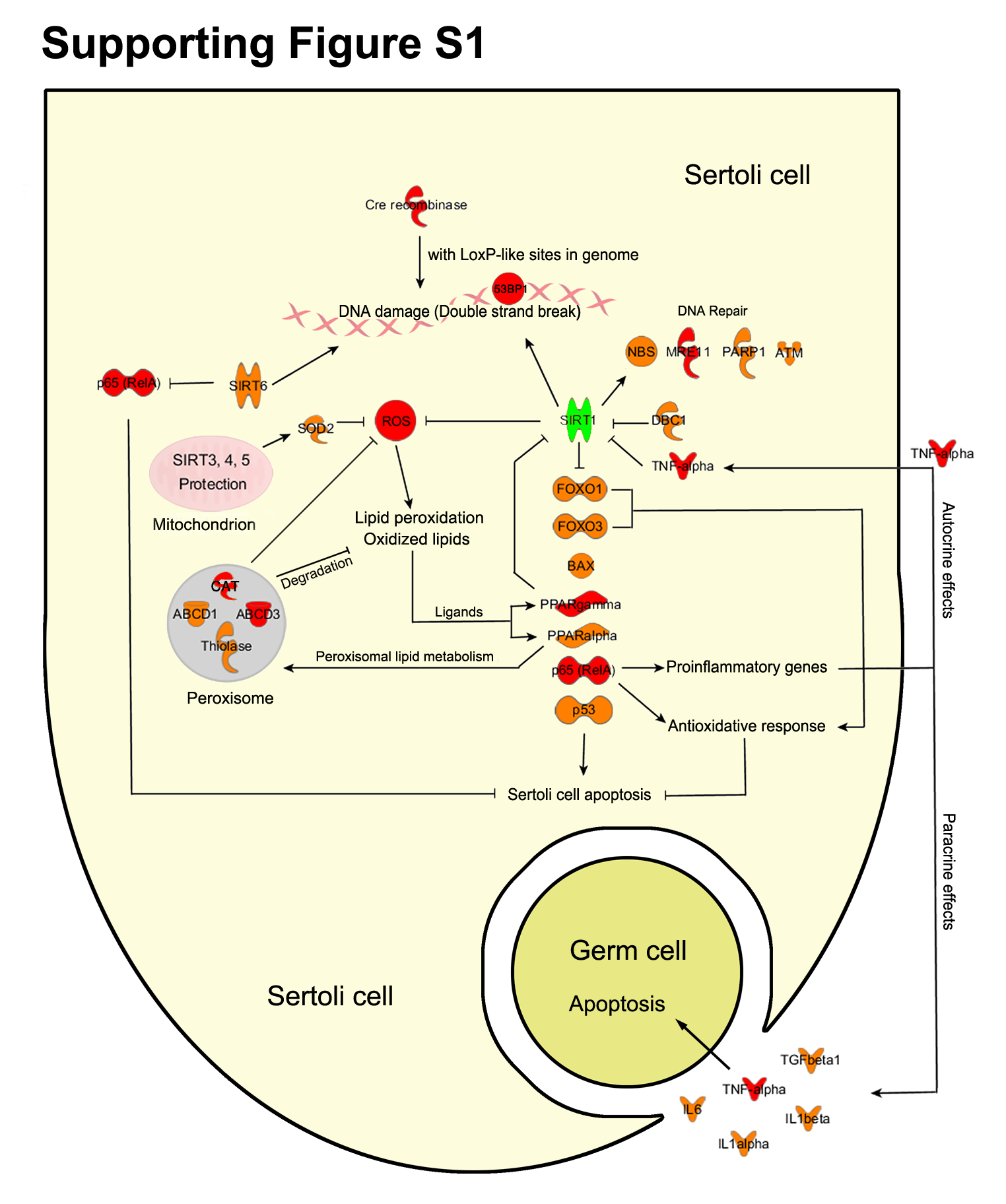

Supplement: Figure S1 — Schematic overview of the model for regulation and transduction of AMH- Cre recombinase induced stress in Sertoli and germ cells. (TIF) [file pone.0041097.s001.tif]

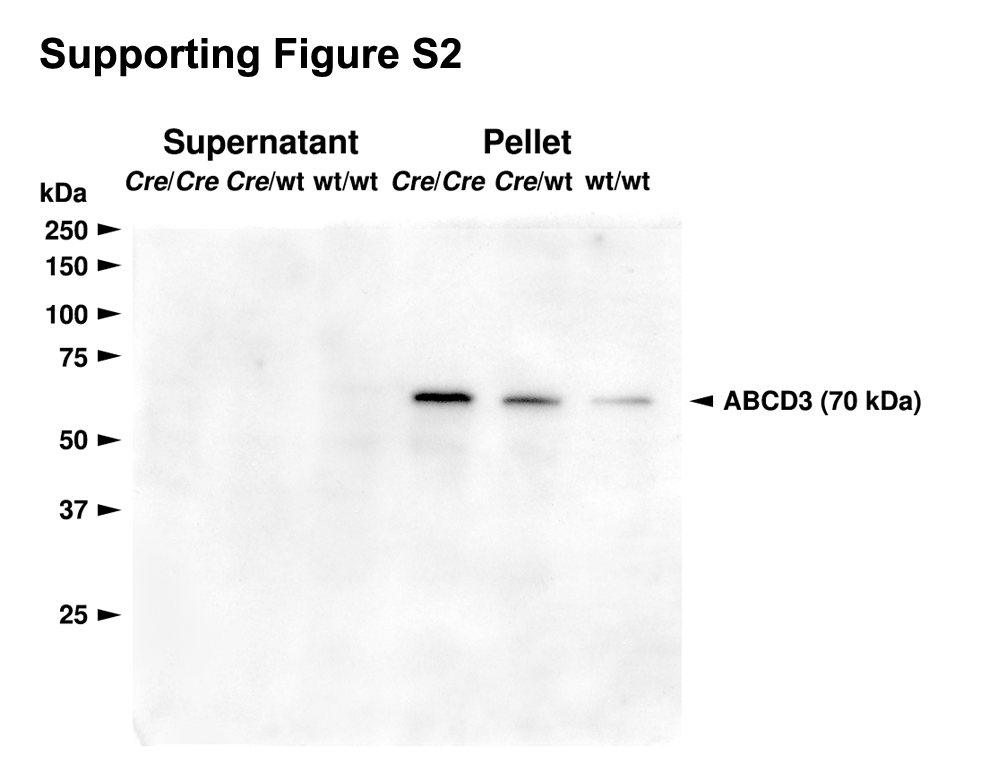

Supplement: Figure S2 — Western blot of cytosolic and enriched organelle fractions of testes of AMH- Cre /AMH- Cre , AMH- Cre /Wt and Wt/Wt mice, probed with an antibody against the peroxisomal membrane lipid transporter ABCD3. The blot shows the high specificity of the antibody, which is necessary to obtain an optimal and reliable staining for the localization of organelles in tissue sections. (TIF) [file pone.0041097.s002.tif]

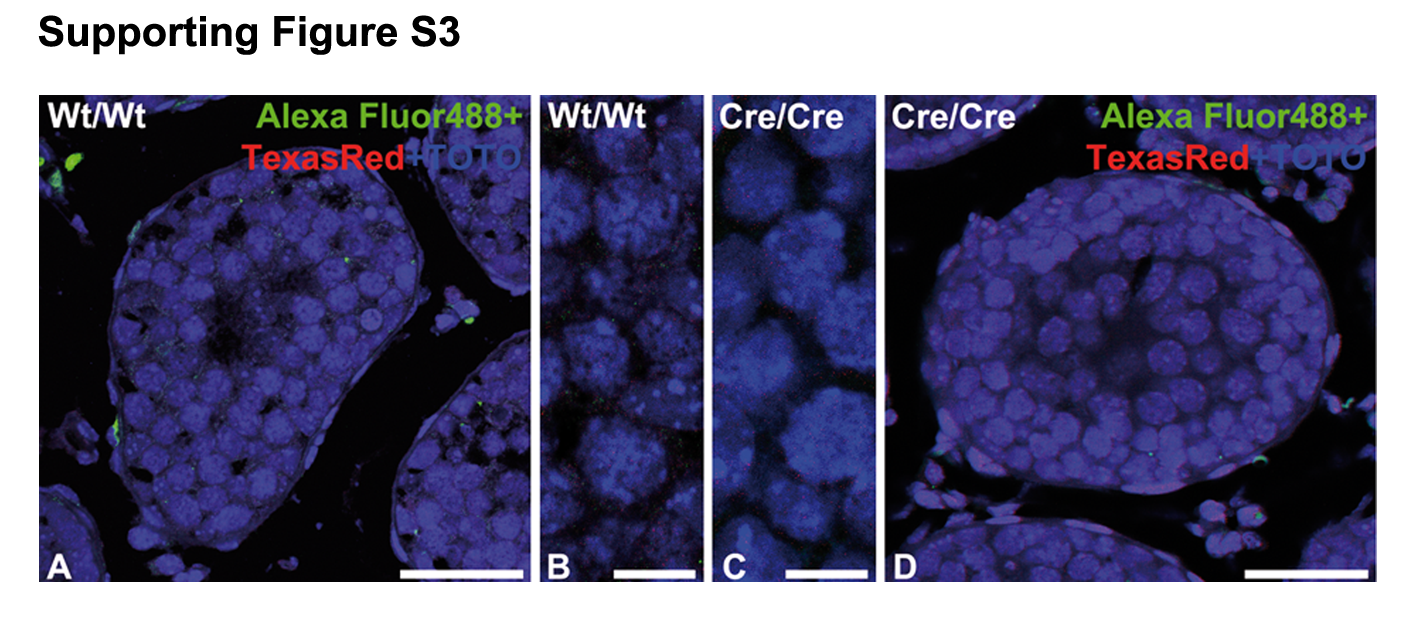

Supplement: Figure S3 — Negative control sections (without primary antibody) of the testes of Wt/Wt and AMH- Cre /AMH- Cre mice, processed in parallel to double-immunofluorescence preparations presented in the manuscript, depicting only very few unspecific binding sites of the secondary antibodies and revealing the high reliability of the immunofluorescence protocol used. (TIF) [file pone.0041097.s003.tif]

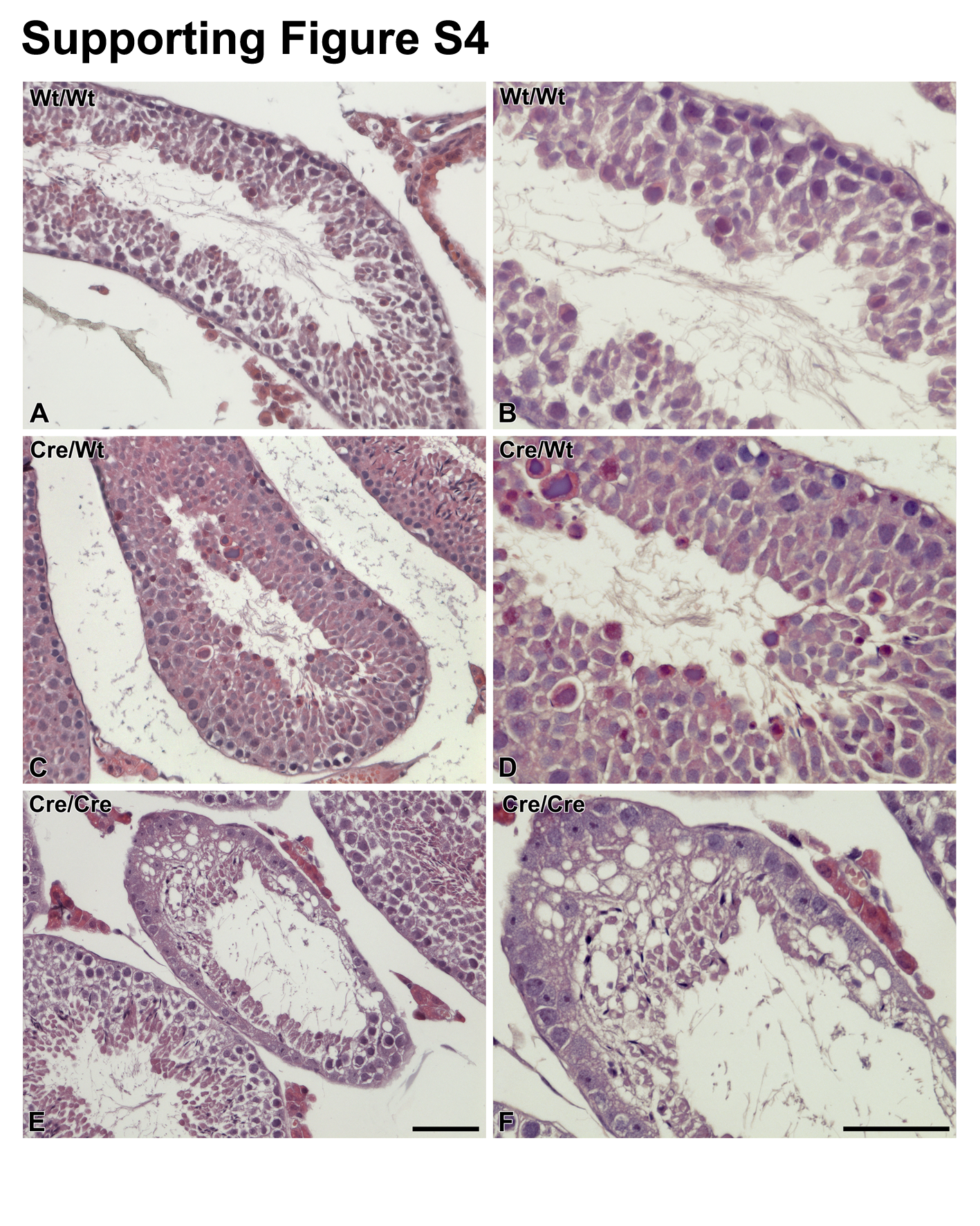

Supplement: Figure S4 — Hematoxylin and eosin (H&E) staining of testis sections of sexually mature adult animals (P120) with Wt/Wt (A, B), AMH- Cre /Wt (C, D) and AMH- Cre /AMH- Cre (E, F) genotypes. A, C, E: lower magnification overviews (bar: 50 µm); B, D, F: corresponding partial higher magnifications of the same regions (bar: 50 µm). Note the higher amount of apoptotic germ cells in a semininiferous tubule (seminiferous epithelium at stage 9) of a heterozygous Cre animal. In homozygous Cre mice also a higher apoptosis rate was noted (in stage 9 tubules, data not shown) and a mosaic pattern was observed, with few degenerated seminiferous tubules exhibiting severely impaired spermatogenesis located besides normal appearing ones with regular spermatogenesis. (TIF) [file pone.0041097.s004.tif]
