# Supplementary material for: Cre-Mediated Stress Affects Sirtuin Expression Levels, Peroxisome Biogenesis and Metabolism, Antioxidant and Proinflammatory Signaling Pathways
Source: PLoS One. 2012 Jul 19;7(7):e41097. doi: 10.1371/journal.pone.0041097 (PMC3400606; doi:10.1371/journal.pone.0041097)
Supplement: Sequence S1 — Assembled sequence obtained by PCR-based genome walking proving the insertion of the transgene in the intron 3 region of the Plekha5 gene on chromosome 6. Sequencing was started in both directions from the AMH-Cre transgene cassette to obtain flanking regions at both sides. (DOC) [file pone.0041097.s005.doc]

**Supporting Sequence S1:**

**PCR-based genome walking from inserted AMH-*Cre* gene to flanking regions of *Mus musculus* strain C57BL/6J**

5’-TTATGAGAAGGGGAGTATGCTGTGTGAAATGACCCAGGTCTCCTGTGGCCTTGAATAAGCCACAGAATGTGGGTCTTCCACCTATCCTATCAAAAATGCTTATGGTAATAAAAGCAACAAAAAATTTCCCTATGTTTCGAGACCCGAGGTTTTTTCCTTTAGTGGTTTGGTAAATAACTCTGGTGTTAAACTTAACACTCAGCGGATCTGGTTTTGGGTGCTATTTTAGTCAATCTTCATAACTGTTGTTTGAGATGGTATCTCTCCGGATCTTTGTATGTAAAACCATTTCTGGGAAGATGTGAACAAACCCAAGTAGGGAACCAAGGACAGACCAAAGCATTCGTCGAAATTCTTGCCAAGCAAAAGAGTGGAAGTGGAGGTGTGGTGAGCCCTGGCACATCCCTCCGGTTAACCTTGAAGCCTTTGAAGGAGGACAGGGAGAAGCGGCTGTTAGAGGCAGCTCCTCCCGGACCCTCAGCAGTGGGAATGGCTAGAGGTGACCCCGGTTCAGAAAGCTGGGGAAGTGACCAGGGTGGAAGATAGAGACTTGTGTCCATAGGGTTCAGGAAGGCGGGCACGCTACCCAGCAAGTGTTCTTTACAGAAGGCGGGGACAGCCTGTGTGCCTGGCCTCCAGACAGAAGCCTGCAGGGCAGAGGTCCCGGGGCAGGTGCACCGGGTGGGCGAGGGTGGCTAGAGCGGCAGGCTCCTGGTGGGGAGGCCCTGACAGGTCCTGGGCTTGGGCTCAGGACGGTGAGGCGGCAGAGGGCTTGTGAGTGTGCGTGTCTGAGGCCGCATGGAGAGTGGCCGGGCGGCAGGCAGCCCAGCAGCCATTCTCGGGGTCCAGGCTGCTGAGCTCTGGCGGGAAGGGCATCCTCTCTCGCTCACCGTATACATGGTGTCAGCCCTGTTCAGGCGGCCCCACTCGAATCCCAGAGCCAGGGTGCCGAGGGCCCCTCCCTACCCAGTGGCCCCAACTCCTGCTGGGTGGTATCCCAGCACCCCTGGTGAAGGGCTCTGGGGATTGGGACAAAGGAGCACCATCCTCGGTCCCTGCCTGAGTGACTCCGCAGGTAGCTCAGCTCTGGCGCACCTGTGCCTTCACCTATACCAGAATCCCAGAGCCTGGGCGGAGATTGTCTAATGGTTGCCAGAGCTGCAGGGCCTCCCCCAGGGTCCCGCTCTCGTTCAGTGGGTGGCATTAGCCTGCCCCAGGTTCCTCAGCTTCGGAGAGGACAAGCAGCCGGGACCTGAGAGCTTTCTGTGTTGCAGTGACCAAGCAGAGAGACTCGGAGATGGGCCAGCAGAGCCTCCTCTTCCAGGTGAGATCAGGGACTTGGGCGTGGGGGGCGGCCAAAGGCACCCAAGAGGCGGCTCTGCCACCCGGCCGTGTCCCTGCAGATTGACTACCCTGAGATCGCCGAGGGCATCATGCCACGTCACCGCTTCATGTCTGCGTACGAGCAGAGGATCGAGCCTCCGGACCGGCGCTGGCAGTACCTGCTCATGGCCGCCGAGCCCTACGAGACCATTGCCTTCAAGGTAGCGTGGCTGCGGGGTTCCCTGGGCCCCCTTGAGATGTGCAAGCCAGAAGGATCTGCATAGGCTGGGCAGGATGCCCCTCCCATCGGGCTTGGGGTCCCCTGGGCCCCCCCCAAGTCGGAGCCAGAAGGATCTGCACAGGCCGGGCAGGATGCCCCTCCCATTGGGCTCTGCAGGAGGGCCTGCTTGGGCTCCACTGGGCCAATGAGAAGGGAGGCCGGCAGGGCTGAGCGCCAGGCCTGTGAGGCCTTTGCCAAGTGACCTCACTTCCTGGGCCAGGCTGCTGGAGGGCTTCCTACAGCCTGTCCCCATGCCTGGACAGGGCGGCCATCAGGAAACAAATAATACAAACGGTGACTGCCCAGGGAGGGGGCCAACAGCGTGCTAGCATTGAGGCCAGGTCTGTTTCACTCCAGAACTGAACCCTGTGCCCCACGAAAGTCTTACCAGCCTTCTCCTGGGCTCCCAGGGTAGAGTCCGGGCTGGGGAGGCTAGTGCCTCGGGCTCCCTGCCTGATGGTCGCCCTGAGGTCACAGGGACCAGGAGCCCTCTCTGTCCCCCGCCCTCCCAGGTGCCGAGCAGAGAGATCGACAAGGCGGAGGGCAAGTTCTGGACACACTGGAACCGGGAGACCAAGCAGGTGAGTGGCTCGCCCCGAGCCTGGCTCCTTCCCACCCAGCCCTGGCGGCCCTAGCCCTGCCCTAGCCCCACCCGTGCCTGCTGAACCTTTCTCCGTCTCTCTAGTTCTTCCTCCAGTTCCACTTTAAGATGGAGAAGCCCCCGGCTCCACCCAGCCTCCCTGCTGGCCCCCCTGGGGTGAAGCGGCCTCCACCCCCGCTGATGAACGGTCTGCCCCCTCGGCCACCGCTGCCTGAGTCTTTGCCACCGCCCCCGCCAGGAGGCCTGCCTCTGCCACCCATGCCCCCCACAGGGCCTGCGCCCTCAGGGCCCCGGGACCACCCCAGCTACCCCCGCCAGCTCCAGGGGTCCACCCCCCGGCCCCAGTGGTGCATCCCCCTGCATCTGGGGTCCATCCCCCAGCTCCTGGCGTCCACCCCCCAGCTCCTGGCGTCCATCCCCCAGCCCCTGGGGTCCACCCACCAACCTCTGGGGTCCACCCCCCAGCTCCTGGAGTCCACCCTCCAGCCCCCGGGGTTCACCCACCAGCCCCCGGAGTCCACCCACCAGCCCCTGGGGTTCACCCACCAGCCCCAGGGGTCCATCCTCCCCCATCAGCGGGGGTTCACCCCCAGGCCCCGGGGGTGCACCCAGCAGCCCCCGCCGTTCACCCTCAGGCCCCAGGGGTGCACCCACCAGCCCCAGGGATGCACCCTCAGGCCCCGGGGGTCCACCCCCAACCTCCCGGGGTCCATCCGTCGGCTCCTGGGGTCCACCCTCAGCCTCCGGGAGTTCACCCCTCAAATCCTGGGGTGCACCCCCCAACTCCCATGCCCCCAATGCTGAGGCCCCCACTTCCCTCCGAAGGCCCAGGGAACATACCTCCCCCTCCCCCAACCAACTGAGAAGCTGCTCCCTCCCCCAGCAAGCCCAGCGCCAGGTGCTCTTGCCTTTTCCCACTGAGAGAAGGCTGCTCTTTTGTACTGCCCCCCGCTCATTAAACAGCCTCCCCCAGCCCTGAGTGCACTGATGTCCGCAGCGCTGCCCTACTGTGTCAGTGTGTGTGGGAGTGCCAGGCACAGCACCATCCCCCAGTTTGGGCCGACTGGGGAGGGCCTGGGGCCCGCCAGGAGACACCTGTGGGAGGCCTGAGAGATGGCTGTACCTTGGAGATGGCCTGGTGGAGGACAGACCCCACCAGCCAGCTAGGAGGGGATCTGGGGTCCTGTTCTGGGGAGGGAAGAGCAGACTCCACGATATCCTTGGGGTCTCCAGATAGCCCACCAGGGGTGGGGAGGGTGAGCAGGGACAGGGCGCCCCCACTGACTTGGGACCCTCCTCCTCCAGGCCCACACCTCAGCACCCAGGACATCTGGGCCCCCCGCCCCCAGCGCTGTCTAGTTTGGTTGCCTGGCCGTCACTCCCAGCCTGGTTCCCACTCCTGTGTCTTCTGGGGATGGCCCTCAAGGACAGCATGTTGACACATCAGGCCCAGCTCTATCACTGGGGAGGGAGATAGGCTGCCAGGGACAGAAAGGGCTCTTTGAGAAGGCCACTCTGCCTGGAGTGGGGGCGCCGGGCACTGTCCCCCAAGGTCGCGGCAGAGGAGATAGGGGTCTGTCCTGCACAAACACCCCACCTTCCACTCGGCTCACTTAAGGCAGGCAGCCCAGCCCCTGGCAGCACCCACGCCTAGTCGACCTCGAGGGGCAGAGCCGATCCTGTACACTTTACTTAAAACCATTATCTGAGTGTGAAATGTCCAATTTACTGACCGTACACCAAAATTTGCCTGCATTACCGGTCGATGCAACGAGTGATGAGGTTCGCAAGAACCTGATGGACATGTTCAGGGATCGCCAGGCGTTTTCTGAGCATACCTGGAAAATGCTTCTGTCCGTTTGCCGGTCGTGGGCGGCATGGTGCAAGTTGAATAACCGGAAATGGTTTCCCGCAGAACCTGAAGATGTTCGCGATTATCTTCTATATCTTCAGGCGCGCGGTCTGGCAGTAAAAACTATCCAGCAACATTTGGGCCAGCTAAACATGCTTCATCGTCGGTCCGGGCTGCCACGACCAAGTGACAGCAATGCTGTTTCACTGGTTATGCGGCGGATCCGAAAAGAAAACGTTGATGCCGGTGAACGTGCAAAACAGGCTCTAGCGTTCGAACGCACTGATTTCGACCAGGTTCGTTCACTCATGGAAAATAGCGATCGCTGCCAGGATATACGTAATCTGGCATTTCTGGGGATTGCTTATAACACCCTGTTACGTATAGCCGAAATTGCCAGGATCAGGGTTAAAGATATCTCACGTACTGACGGTGGGAGAATGTTAATCCATATTGGCAGAACGAAAACGCTGGTTAGCACCGCAGGTGTAGAGAAGGCACTTAGCCTGGGGGTAACTAAACTGGTCGAGCGATGGATTTCCGTCTCTGGTGTAGCTGATGATCCGAATAACTACCTGTTTTGCCGGGTCAGAAAAAATGGTGTTGCCGCGCCATCTGCCACCAGCCAGCTATCAACTCGCGCCCTGGAAGGGATTTTTGAAGCAACTCATCGATTGATTTACGGCGCTAAGGATGACTCTGGTCAGAGATACCTGGCCTGGTCTGGACACAGTGCCCGTGTCGGAGCCGCGCGAGATATGGCCCGCGCTGGAGTTTCAATACCGGAGATCATGCAAGCTGGTGGCTGGACCAATGTAAATATTGTCATGAACTATATCCGTAACCTGGATAGTGAAACAGGGGCAATGGTGCGCCTGCTGGAAGATGGCGATTAGCCATTAACGCGTAAATGATTGCTATAATTATTTGATATTTATGGTGACATATGAGAAAGGATTTCAACATCGACGGAAAATATGTAGTGCTGTCTGTAAGCACTAATATTCAGTCGCCAGCCGTCATTGTCACTGTAAAGCTGAGCGATAGAATGCCTGATATTGACTCAATATCCGTTGCGTTTCCTGTCAAAAGTATGCGTAGTGCTGAACATTTCGTGATGAATGCCACCGAGGAAGAAGCACGGCGCGGTTTTGCTAAAGTGATGTCTGAGTTTGGCGAACTCTTGGGTAAGGTTGGAATTGTCGAGGGATCTCGGAATGGACCCCAACTGCTCCTGCTCCACCGGTAAGACTCCCGATCCTTGGTCTTTAGAATACCAAGTTGGGACCGCAGAGCGGAATCCCCGAGTTGTAGAGGCTTGGCGGGAATAGGCACCTTTAGTTGGCGATTCATTCCGGTTCTTTCTAGAATCCGCTCTTGCAAAAGCCTTCATTAGTTACGAGTATTGTCGAACGGGTCCTTTGGCGGGGTTGGGGCTAGGATTTAGACGCGCAAATGTCCGGTTCCTGATCACCCAGTTAGTGGGGACATCTGGGTTGAGTCCCAGGCATTACTAAACTTACTGTGAATTGCTTGAATTAAGAAAGAGGTGAAGGACCTTTATGTCTTGGGACTCAAAGACATAATCCCTGACTTAACCTGTGAGGAGAAAAGTGGGGCTAGGCTCCCTGCAGCTCCGAGGAGGACTTAGTGAACTGAGCCGGGACTCGTGGTGTTGGCCACTGCTGTAATGCTGCCTCCCTCATGCTGTCTTCTTTCTCCTCCCCAGGCGGCTCCTGCACTTGCACCAGCTCCTGCGCCTGCAAGAACTGCAAGTGCACCTCCTGCAAGAAGAGTGAGTTGGGACACCTTGGGTGGCGGCTAAGGCTAGGGGCGGGGAACTCCTACAAAACTGGCTCTGAGAAATGTCCTTTGCTTCCCGGAGGCCATTGTATTGTCTCGGGGACAGAACTATACAGAGAACTATTTAAAAAAACCGAGGTCTTCTCTGTTGGGGACAGGAAGCAGAGGTCTTCAGCCAGGCTGACCTCTTCCTCCTCCTTTCTAGGCTGCTGCTCCTGCTGTCCCGTGGGCTGCTCCAAATGTGCCCAGGGCTGTGTCTGCAAAGGCGCCGCGGACAAGTGCACGTGCTGTGCCTGATGTGACGAACAGCGCTGCCACCACGTGTAAATAGTATCGGACCAACCCAGCGTCTTCCTATACAGTTCCACCCTGTTTACTAAACCCCCGTTTTCTACCGAGTACGTGAATAATAAAAGCCTGTTTGAGTCTAACTCTGGTTTTCTTGGTGTGGTTTGGCAATAAGAAACTGGGGTGACTTGATAGTCTGGGGATCTGGTTTTGGACCCCCTCGTGCCTTTACCTCCCGCCTCTGGCCCTCACAGAGGGGTAATGTCTTTGGGTAAAGCCAAGCTATCCCATAAGCTTCCTCATGGAAAACAGACCAGCCCGGGCCGTCGACCACGCGTGCCCTATAGTGAGTCATATTACATGTGCTTCAGCTTCTGGGAAGGGAAGGAGCAGGAGGAGGAGCGCGGTGTTAGCGGTTGATCAGAAAGCAGAAGCCTGGAATGAAGAGCAGAGCACCGCTTCCTTAGTTAGCAAAAGACTGGTGAGTGACTGCTTTGTCTGTTTCCAGTAGGCTGTGCCTTTACCTCCGCCCGCAGCTCGGCTGTGCAGAGCCGACAAGGCTGTGTGCAGTTTGTACACAGCCCTGGAGAAATTTGCTCAGCACTCTTTAGTCTCTTCCTTCAGAAATATTACCAGCGCAGTTTCGGGGTGACAGACTCACAAAGTCCTATGATGATAATGCTTCCCATGGCTTCCGCCAATGTCGTTGCTCCTGTGCTGAGTAGGGCTGGCAGAATTGTATGCTGAGAGACCAGGATTTGTTCATCCAGGCAGCTTGGCATCCTTGCAGTCACTGCTCTTGCTCGGCTGCCTTTACCGGTCTATACTGCATTTCCTAAAGTAACTGCAAATACAGACCAGCCCGGGCCGTCGACCACGCGTGCCCTATAGTGAGTCGTATTACAAAA

-3'
