# Supplementary material for: Cre-Mediated Stress Affects Sirtuin Expression Levels, Peroxisome Biogenesis and Metabolism, Antioxidant and Proinflammatory Signaling Pathways
Source: PLoS One. 2012 Jul 19;7(7):e41097. doi: 10.1371/journal.pone.0041097 (PMC3400606; doi:10.1371/journal.pone.0041097)
Supplement: Table S1 — Top 10 ranking of gene and canonical pathway alterations in primary Cre -expressing Sertoli cells (AMH- Cre /Wt versus Wt/Wt) obtained by Ingenuity Pathway Analysis (IPA® software). (DOC) [file pone.0041097.s006.doc]

**Supporting Table S1:**

**Top 10 ranking of gene and canonical pathway alterations in primary *Cre*-expressing Sertoli cells (AMH-*Cre*/WT versus WT/WT) obtained by Ingenuity Pathway Analysis**

**A:** Top 10 ranking of gene alterations

| **Top 10 up-regulated genes** | | **Top 10 down-regulated genes** | |
| --- | --- | --- | --- |
| **Gene abbreviation** | **Value (fold change)** | **Geneabbreviation** | **Value (fold change)** |
| AGPAT6 | 5.043 | S100A9 | -4.825 |
| RSF1 | 3.972 | FZD9 | -4.382 |
| STAT1 | 3.327 | SEPT12 | -4.203 |
| GCNT2 | 3.294 | DAG1 | -4.187 |
| RGS18 | 3.271 | UBQLN1 | -3.684 |
| BACH2 | 3.264 | SUMO1 | -3.651 |
| INSR | 3.206 | MS4A1 | -3.540 |
| DMTF1 | 3.201 | FARS2 | -3.467 |
| GNGT1 | 3.140 | NETO1 | -3.353 |
| CFB | 3.068 | CAPN3 | -3.249 |

**B:** Top 10 ranking of pathway alterations

| **Top 10 IPA Signaling Pathways** |
| --- |
| 1. Interferon Signaling |
| 1. Activation of IRF by Cytosolic Pattern Recognition Receptors |
| 1. PKCθ Signaling in T Lymphocytes |
| 1. Antigen Presentation Pathway |
| 1. GNRH Signaling |
| 1. Role of JAK family kinases in IL-6-type Cytokine Signaling |
| 1. Calcium-induced T Lymphocyte Apoptosis |
| 1. TNFR2 Signaling |
| 1. Role of Pattern Recognition Receptors in Recognition of Bacteria and Viruses |
| 1. Role of NFAT in Cardiac Hypertrophy |
